# Isoform-level profiling of m^6^A epitranscriptomic signatures in human brain

**DOI:** 10.1126/sciadv.adp0783

**Published:** 2025-08-08

**Authors:** Josie Gleeson, Sachithrani U. Madugalle, Ching Yin Wan, Catriona McLean, Timothy W. Bredy, Ricardo De Paoli-Iseppi, Michael B. Clark

**Affiliations:** ^1^ Department of Anatomy and Physiology, The University of Melbourne, Parkville, VIC, Australia.; ^2^ Queensland Brain Institute, The University of Queensland, Brisbane, QLD, Australia.; ^3^ Department of Anatomical Pathology, Alfred Health, Melbourne, VIC, Australia.; ^4^ Victorian Brain Bank, The Florey, Parkville, VIC, Australia.

## Abstract

The RNA modification N6-methyladenosine (m^6^A) is highly abundant in human brain and implicated in neurological disorders. Profiling m^6^A within RNA isoforms is a critical step toward understanding the complex mechanisms that underpin brain function and disease; however, we lack an isoform-level atlas of m^6^A sites in the brain. We applied Oxford Nanopore direct RNA sequencing (DRS) to three postmortem human brain regions—prefrontal cortex, caudate nucleus, and cerebellum—to simultaneously investigate the transcriptome and epitranscriptome at the isoform level. We identified 57,000 m^6^A sites within 15,000 isoforms, revealing both isoform- and brain region–specific patterning of m^6^A modifications. The prefrontal cortex exhibited a distinctive profile of specifically modified isoforms enriched in excitatory neurons and had the highest proportion of unannotated m^6^A sites. A population of isoforms were hypermodified and associated with excitatory neurons in all brain regions. Our results demonstrate the utility of isoform-level profiling of RNA modifications and provide insights into brain region specificity with implications for development and disease.

## Teaser

Direct RNA-seq in brain reveals RNA isoform and region-specific m6A modifications, highlighting their role in gene regulation.

## INTRODUCTION

Complex mechanisms of gene regulation are critical for the unique functioning and development of the human brain. A single gene can produce multiple RNA isoforms through alternative splicing and polyadenylation processes, greatly expanding the transcriptional diversity of both protein-coding and noncoding RNAs (*1*). Different gene isoforms commonly have distinct posttranscriptional fates and can encode RNAs and protein products with varying or opposing functions (*2*, *3*). The brain has the highest levels of splicing activity in human tissues, and various neuronal pathways are regulated by differential expression of isoforms, such as cell fate determination, axon guidance, and synaptogenesis (*4*).

Posttranscriptional chemical modifications can also regulate the function of protein-coding and noncoding RNAs. The most abundant internal mRNA modification in eukaryotes is N6-methyladenosine (m^6^A), which regulates many aspects of the brain transcriptome (*5*–*8*). The brain has the highest levels of m^6^A in human tissues, which increases from developmental stages into adulthood (*5*, *9*). The dysregulation of RNA modification processes has been implicated in many neurodegenerative and neuropsychiatric disorders (*10*), and m^6^A is critical for brain development, learning, and memory (*11*, *12*).

Because of the established importance of differential isoform expression (DIE) in the human brain, it is essential to characterize m^6^A modification sites at the isoform level. Popular methods to study m^6^A involve immunoprecipitation of modified RNA fragments followed by short-read sequencing (SRS) (*5*, *6*). However, these methods only provide information on m^6^A modifications at the gene level. The exact nucleotide position and stoichiometry of m^6^A sites cannot be determined using these methods, and it is therefore often impossible to identify which original RNA isoform contained the modification (*13*, *14*). Chemical-based and enzyme-based detection methods that induce mutations at modification sites enable the detection of m^6^A at single nucleotides (*15*, *16*). However, these techniques have had limited uptake as they do not provide isoform resolution and require complicated and expensive protocols. Therefore, there is a lack of knowledge about how m^6^A modifications are regulated at the isoform level, and it remains unknown whether isoforms are differentially modified within genes or between tissues.

Long-read direct RNA sequencing (DRS) from Oxford Nanopore Technologies (ONT) addresses many of these limitations by providing single-nucleotide isoform-level resolution of m^6^A modifications. DRS enables RNA sequencing without fragmentation or conversion to cDNA, preserving RNA modifications and polyadenylated (polyA) tail lengths. In addition, the quantification of m^6^A modification rates with DRS is highly similar to that of enzymatic approaches (*16*–*18*). However, no studies have applied DRS to the human brain to investigate the critical role of m^6^A modifications in this complex organ.

We aimed to characterize isoform-level m^6^A modification sites across the human brain transcriptome and integrate this with both isoform expression and poly(A) tail lengths in different brain regions. To our knowledge, we have performed the first application of DRS to the human brain, profiling tissues from three functionally distinct regions: prefrontal cortex (PFC), caudate nucleus (CN), and cerebellum (CB). We provide an isoform-level transcriptome-wide map of m^6^A modification sites and identify widespread changes in isoform expression, m^6^A profiles and poly(A) lengths both between gene isoforms and between the different brain regions. Our study reveals brain region–specific regulation of m^6^A modifications within isoforms and shows that many specifically modified isoforms are associated with distinct cell types in different brain regions. We show that modification rates of m^6^A sites in different isoforms from a single gene are influenced mainly by isoform structure and proximity to downstream exon boundaries. In addition, we have created a web app to explore and visualize the data: https://clarklaboratory.shinyapps.io/human_brain_m6a/. On the basis of our findings, we recommend that m^6^A modifications be interpreted in isoform- and tissue-specific contexts.

## RESULTS

### Long-read DRS of human brain samples

**Fig. 1.**
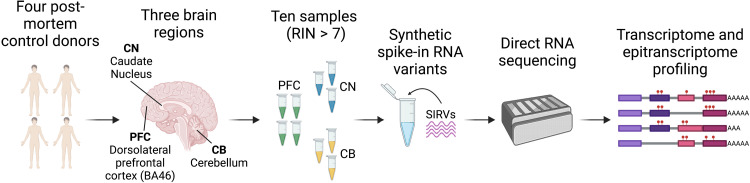
Experimental overview of DRS of post-mortem human brain samples. RNA was isolated from brain tissue of donors without neurological disorders from: PFC, CN, and CB (table S2). Samples with an RNA integrity number (RIN) >7 were sequenced using ONT’s PromethION device. SIRVs were added as controls.

We applied DRS to postmortem human brain samples from three brain regions: PFC, CN, and CB (Fig. 1). DRS generated >52 million high-quality reads (*q* score > 7) from 10 samples with a median read length of 720 nt (table S1). We included synthetic spike-in RNA variant (SIRV) RNAs as a control and sequenced 360,695 SIRV reads. We identified the expression of >22,000 genes and >62,000 isoforms across the brain regions, and the reads covered a median of 59.50% of their mapped transcript isoform with a median accuracy of 90.82% (fig. S1).

### Identification of brain region–specific transcriptional patterns and isoform switches

**Fig. 2.**
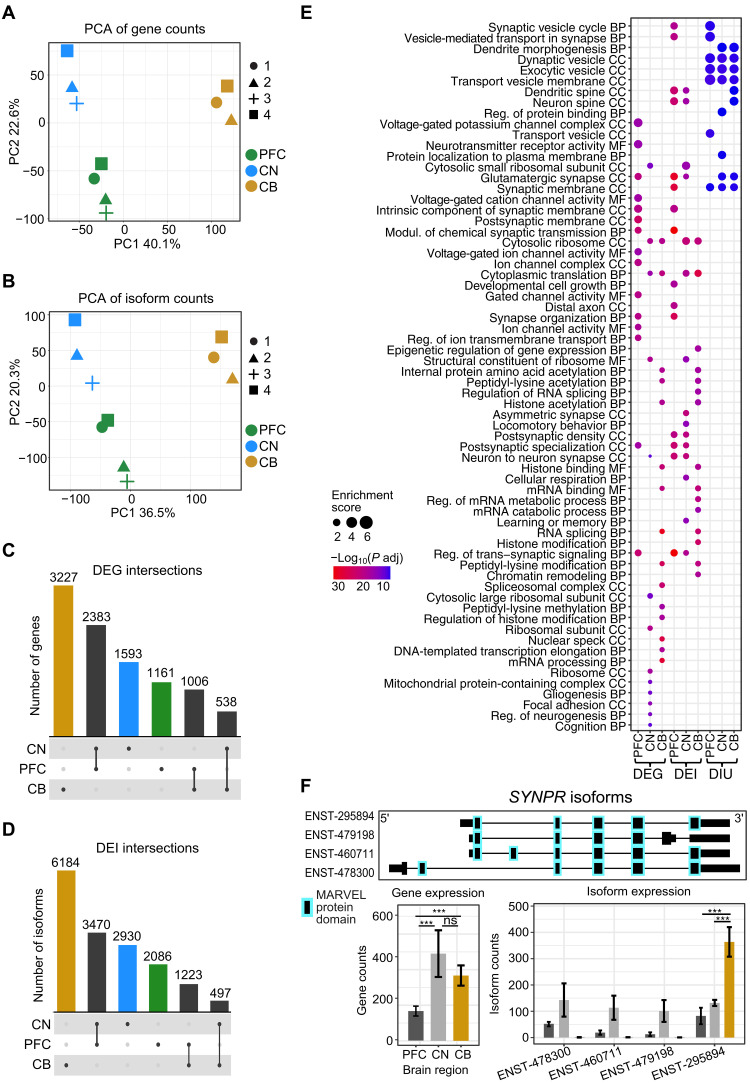
Widespread gene and isoform expression differences between brain regions. Principal components analysis (PCA) of (**A**) gene counts and (**B**) isoform counts from human brain samples. Colors indicate brain regions (PFC = green, CN = blue, CB = yellow), and shapes indicate individual donors. (**C**) UpSet plot of DEGs between brain regions. (**D**) UpSet plot of DEIs between brain regions. (**E**) Gene Ontology (GO) analysis of DEGs, DEIs, and genes with DIU in each brain region. (**F**) *SYNPR* gene and isoform expression plot. Ensembl transcript (ENST) IDs omit five 0s for brevity. Top panel shows the isoform structure with protein domains highlighted. Gene and isoform expression bar plots are shown below. In CB, only one isoform (ENST00000295894) was expressed, whereas in PFC and CN, the gene expression is composed of multiple isoforms. Significance of adjusted *P* values is indicated by “***” for <0.001. ns, not significant.

We explored expression differences between the brain regions and found that samples clustered by brain region for both gene and isoform expression, with CB having the most distinct expression profile compared to both PFC and CN (Fig. 2, A and B). We found ~10,000 (*n* = 9908) differentially expressed genes (DEGs) between the brain regions (Fig. 2C, Table 1, and table S3), many of which confirmed previous observations of genes known to be up-regulated in particular brain regions, such as the increased expression of *DRD2* in CN (*19*).

**Table 1.** Genes and isoforms with significantly up-regulated expression or usage in each brain region. Significance = adjusted *P* value < 0.05.

	DEG	DEI	DEI, no DEG	Gene with DIU	DIU	Gene with DIU, no DEG	DIU, no DEI
**PFC**	4550	6779	1328 (19.59%)	199	251	121 (60.80%)	71 (28.29%)
**CN**	4514	6897	1647 (23.88%)	300	488	214 (71.33%)	132 (27.05%)
**CB**	4771	7904	2029 (25.67%)	310	346	199 (64.19%)	83 (23.99%)

We also identified 16,390 differentially expressed isoforms (DEIs) between brain regions (Table 1 and table S3), and most DEIs displayed brain region–specific up-regulation. Isoforms up-regulated specifically in CB were the largest category of DEIs, followed by those up-regulated in both PFC and CN (Fig. 2D). For example, the gene *SYNPR* encodes a synaptic vesicle component, synaptoporin, and was expressed in all three brain regions. However, only one isoform contributed to the expression profile in CB, whereas four isoforms were expressed in PFC and CN (Fig. 2F).

Changes in the proportion each isoform contributes to gene expression between tissues are also biologically relevant and were examined with a differential isoform usage (DIU) analysis. The results of DIE may largely reflect those of gene expression, which can mask complexity at the level of isoform usage. We found 764 isoforms encoded by 317 genes with differential usage between brain regions (adjusted *P* value < 0.05 and proportion change > 0.2) (Table 1 and table S3). Of the features with DIU in each brain region, 26% of the isoforms and 65% of the genes did not have up-regulated differential expression (DE), highlighting the additional insight provided with DIU. Gene Ontology (GO) analysis of genes with DIU identified a markedly different profile to DEGs or DEIs (Fig. 2E and table S4). The genes with DIU displayed a consistent signal for synapses and synaptic vesicles, suggesting a specific gene regulatory program for these genes involving isoform switching.

**Fig. 3.**
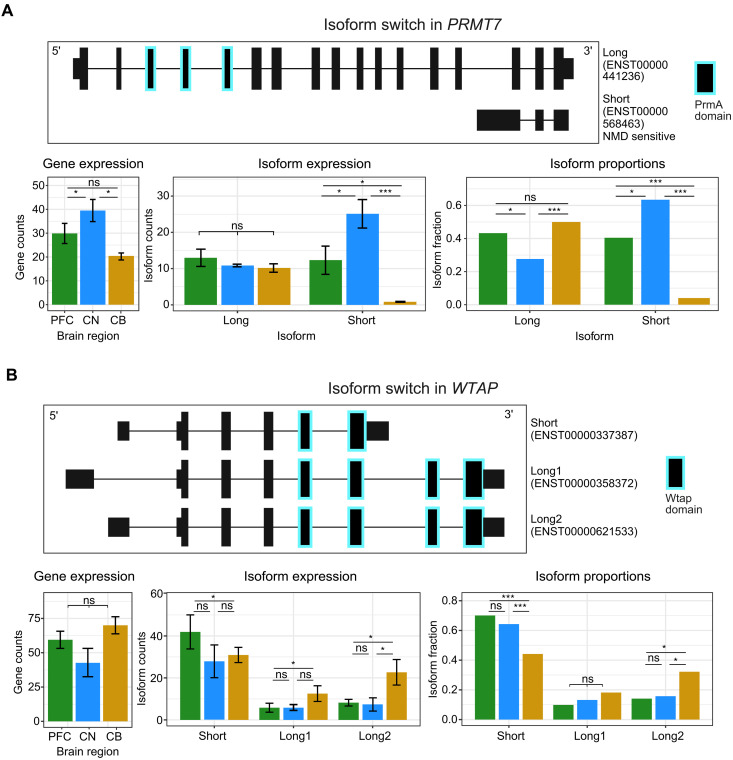
Clinically relevant genes exhibit different isoform usage between brain regions. Switch plots for isoforms with differential usage from (**A**) *PRMT7* and (**B**) *WTAP*. Top of each panel shows the isoform structures with protein domains highlighted. Gene expression, isoform expression, and isoform proportions are shown as bar plots below for PFC (green), CN (blue), and CB (yellow). Significance of adjusted *P* values is indicated by * for <0.05 and *** for <0.001. ns, not significant.

We identified several genes implicated in neurodevelopmental and neuropsychiatric conditions that exhibited DIU, including *PRMT7*, *RBFOX1*, and *GRIA1* (*20*, *21*). The overall expression of *PRMT7* mRNA was highest in CN (Fig. 3A). However, protein expression data from the Human Protein Atlas identified the opposite result (*22*). *PRMT7* DIU analysis revealed that PFC and CB both expressed a greater proportion of the canonical protein-coding isoform compared with CN and that most of the gene expression in CN was due to the expression of a shorter, noncoding nonsense-mediated decay (NMD) isoform. This result highlights how expression at the gene level can mask underlying complexity at the isoform level.

The *WTAP* gene, a subunit of the m^6^A writer complex, had an isoform switch in CB compared to PFC and CN (Fig. 3B). WTAP interacts with METTL3, METTL14, and VIRMA to control m^6^A modification levels on RNA. Most of the expression in CB was from two longer isoforms that contained the complete WTAP protein domain. In contrast, PFC and CN primarily expressed a short WTAP isoform missing two exons required for the WTAP protein to bind VIRMA (*23*, *24*). Despite no significant change at the gene expression level, this isoform switch may result in decreased activity of the m^6^A writer complex in PFC and CN.

### Isoform-level map of m^6^A modification sites in the human brain

DRS enables the identification of m^6^A modification sites at the isoform level with single-nucleotide resolution, allowing us to determine the exact transcriptomic position of a modification and the modification rate (proportion of modified reads) at these sites using m6anet (*18*). We detected 1.14 million DRACH sites that were tested for m^6^A modification, identifying 73,843 sites with an m^6^A modification probability of >0.9. We further filtered these for sites reported as modified in >1 sample, resulting in 57,144 high-confidence m^6^A sites (Materials and Methods and table S5). All downstream analysis was performed on these high-confidence sites. We also tested for m^6^A modifications within the unmodified SIRV control reads. The SIRV transcriptome contains 1750 DRACH sites, none of which were identified as m^6^A modified (probability of >0.9) in any sample, indicating a low level of false-positive m^6^A sites in our data.

**Fig. 4.**
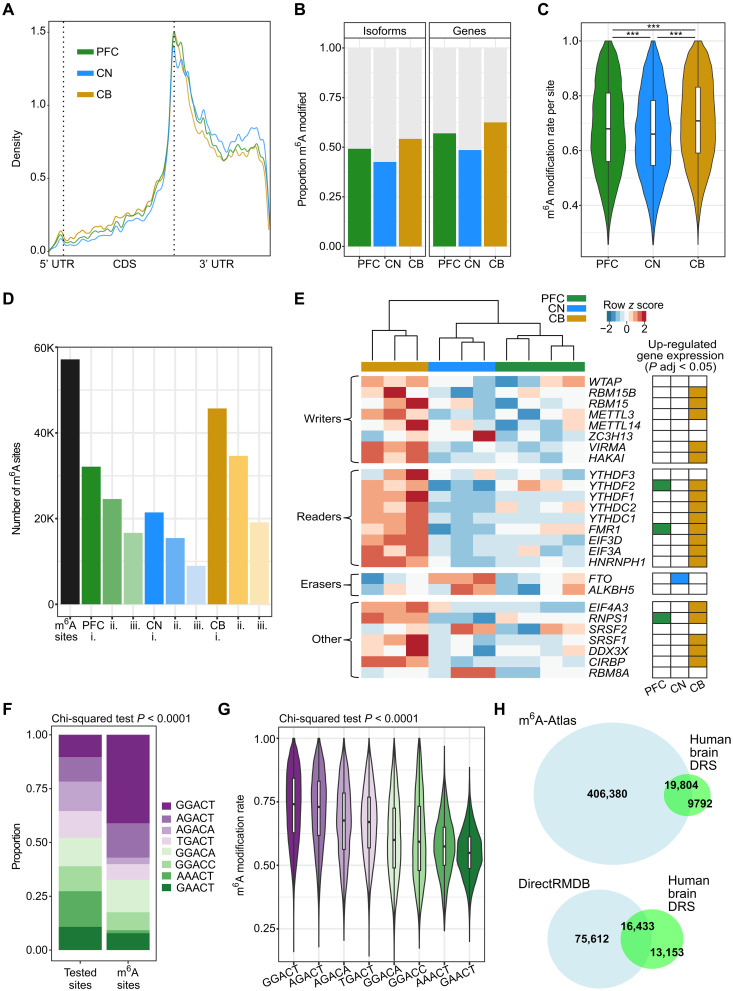
Isoform-level m^6^A modification sites in three human brain regions. (**A**) Metagene plot showing the distribution of m^6^A modification sites along mRNA isoforms for PFC, CN, and CB. (**B**) Proportion of modified genes and isoforms in each brain region. (**C**) Modification rate per m^6^A site in each brain region. (**D**) Number of m^6^A sites detected in >1 brain sample (*n* = 57,144) (black). m^6^A sites per brain region detected in i. at least one sample, ii. at least two samples, and iii. at least three samples. (**E**) Heatmap of gene expression of m^6^A-related genes across three brain regions. (**F**) Proportions of each DRACH motif in all tested sites and m^6^A sites. (**G**) Modification rates per DRACH motif for all m^6^A sites. (**H**) Venn diagrams showing intersections of m^6^A sites identified in our human brain DRS data compared with those identified in either m^6^A-Atlas (top) or DirectRMDB (bottom) (*30*, *31*).

The high-confidence m^6^A sites followed a typical distribution with enrichment around stop codons and in 3′ untranslated regions (3′UTRs) (*5*, *6*) (Fig. 4A). In our dataset, quality control (QC) analyses did not show a relationship between modification rates and sample postmortem interval (PMI), individual donors, donor age, sex, or sample batch (fig. S2). However, larger sample sizes would be required to comprehensively investigate any effect these factors may have on observed brain m^6^A levels.

There were 15,368 isoforms modified with m^6^A across the brain regions and a mean of three m^6^A sites per isoform, consistent with previous studies (*5*, *6*). More than half of the total detected genes (65%, *n* = 7389) and isoforms (55%) were modified with m^6^A (Fig. 4B), and m^6^A sites had a median modification rate of 0.66 (Fig. 4C). We estimated that at least 27% of the reads tested for modifications contained at least one m^6^A site. These results demonstrate that many mRNA molecules are m^6^A modified in human brain tissue and that these sites are commonly found with high stoichiometry.

We found that 25,887 m^6^A sites (45%) were consistently identified in at least three samples per brain region (Fig. 4D). To investigate the impact of read coverage and stoichiometry on m^6^A site discovery, we compared the number of supporting reads and modification rate of m^6^A sites detected in two or less samples (lower reproducibility) versus at least three samples (higher reproducibility) per brain region. We found that the latter had significantly higher coverage [mean read coverage increase >15 reads, Mann-Whitney *U* (MWU)–Wilcoxon, *P* < 0.0001] and modification rates (mean rate increase >20%, MWU *P* < 0.0001) (fig. S3, A and B). Therefore, the read coverage and modification rates both affect the reproducibility of m^6^A sites between samples, and higher reproducibility would be obtained with higher read depths per sample. We also found that the modification rates at m^6^A sites were highly correlated between samples from the same brain region (fig. S3C).

Cerebellum had the highest percentage of modified genes (63%, *n* = 6267) and isoforms (54%, *n* = 13,720) and the highest median modification rate (0.71), consistent with previous studies of CB in mice (*25*, *26*). The expression of multiple genes encoding m^6^A writers and readers was significantly higher in CB, which may account for the increased levels of m^6^A modification observed in this brain region, while the m^6^A eraser *FTO* was significantly up-regulated in CN (Fig. 4E and table S6). Notably, samples clustered by brain region even when subsetting expression to only m^6^A-related genes, suggesting that brain region differences in m^6^A profiles may partly be due to region-specific regulation of the m^6^A machinery.

It has been previously observed that high m^6^A modification levels are associated with long 3′UTRs of isoforms (*27*, *28*). We investigated whether isoforms in our data had changes in 3′UTR lengths in the different brain regions that were contributing to some of the differences in m^6^A modification levels. We compared both the total isoform lengths and 3′UTR lengths between each brain region and found no differences in these features in all detected isoforms (counts >5). However, when we compared the isoforms that were up-regulated (DIE) or m^6^A-modified in each brain region, there were significant differences in both the total length and 3′UTR lengths between brain regions (fig. S4, A to C). CB had longer isoform and 3′UTR lengths than CN in both cases, consistent with the increased modification levels observed in CB. Therefore, the up-regulation of longer isoforms in CB may partially drive the increased levels of m^6^A in CB, and the differences in modification levels observed between brain regions are, in part, a consequence of tissue-specific isoform expression patterns.

The examination of DRACH motifs revealed that “GGACT” was the most commonly modified motif, significantly enriched compared to its abundance within RNA, while GGACT and “AGACT” had the highest modification rates (Fig. 4, F and G) (*16*, *29*). No correlation was observed between the modification rate at m^6^A sites and the motif frequency in m^6^A sites. All brain regions harbored similar proportions of each DRACH motif, and the m^6^A sites in CN had consistently lower modification rates compared with the other brain regions overall and within each DRACH motif (Fig. 4C and fig. S4D). Therefore, we expect that the lower m^6^A levels observed in CN are likely due to differences in the expression of m^6^A-related genes and the expression of particular isoforms rather than a bias toward specific motifs.

### Discovery of previously unannotated m^6^A modification sites

We compared our gene-level m^6^A modification sites (*n* = 29,596) with those previously annotated in two m^6^A databases, m^6^A-Atlas or DirectRMDB (*30*, *31*), and found that 71.36% of the sites in our data had been previously annotated in human tissues or cell lines (Fig. 4H and fig. S5A). The unannotated sites in our data had only marginally lower modification probabilities (−0.0159, *P* < 0.0001) and rates (−0.0021, *P* < 0.0001) compared with the annotated sites. We found that long noncoding RNA (lncRNA) biotypes were enriched in unannotated sites in our data and DirectRMBD sites compared with those in m^6^A-Atlas (*P* < 0.0001), suggesting that DRS may be a more suitable technique for identifying m^6^A within lncRNAs than previous methods (fig. S5B).

Although CB had the highest total number of genomic m^6^A sites (*n* = 24,054), PFC had the highest percentage of unannotated m^6^A sites (26.05%, *n* = 4,438) and the highest total and percentage of genes found with only unannotated modification sites (*n* = 1018, 17.41%). Hence, while the CB displayed a higher frequency of modification, the PFC exhibited a less annotated and more distinctive m^6^A modification profile. Genes not previously identified as m^6^A modified were associated with brain-specific GO terms such as “regulation of synaptic plasticity,” “synaptic signaling,” and “behavior” (table S7). In contrast, genes with only known sites were associated with more general terms such as “RNA splicing,” “protein catabolic process,” and “cellular protein localisation,” which highlights the additional information on m^6^A modifications that DRS can provide, and the existence of previously unidentified m^6^A modifications on key brain genes (table S7).

### Common and brain region–specific m^6^A modification of RNA isoforms

**Fig. 5.**
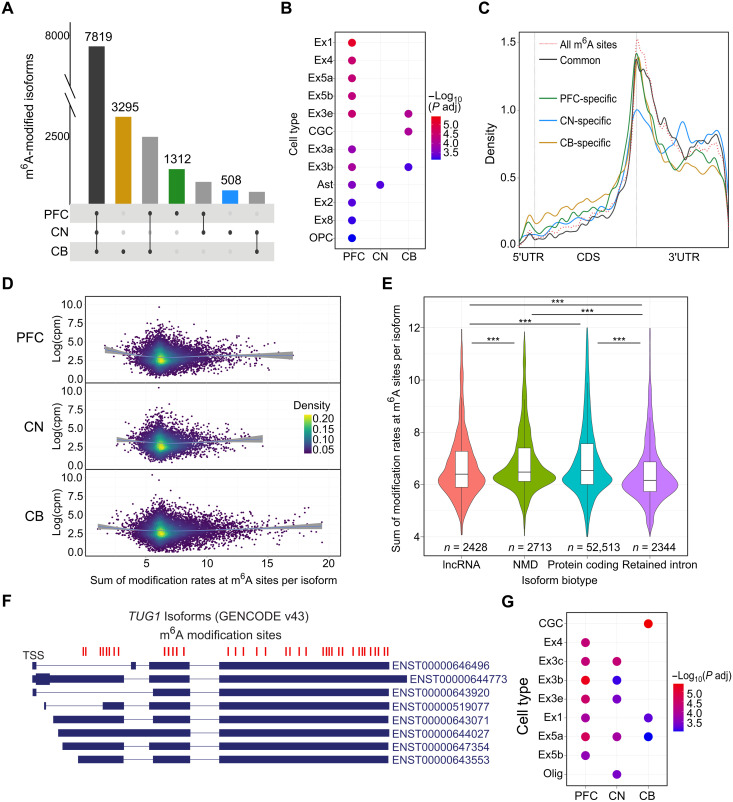
Brain-region-specific m^6^A modification patterns. (**A**) Isoforms with common and specific m^6^A modification in each brain region. (**B**) Cell type–specific analysis of the genes encoding isoforms with specific m^6^A modification (and no expression up-regulation) in each brain region. Ex, excitatory neuron; CGC, cerebellar granule cell; OPC, oligodendrocyte precursor cell; Ast, astrocyte. (**C**) Metagene plot showing the distribution of m^6^A modification sites specific to each brain region and common to all brain regions. The background of all m^6^A modification sites is shown with the dashed red line. (**D**) Scatter plots colored by density for expression in log(counts per million) per isoform compared to summed modification rates at m^6^A sites per isoform for each brain region. (**E**) Summed modification rates at m^6^A sites per isoform plotted per transcript isoform biotype. Only the significant MWU comparisons are labeled. Significance of adjusted *P* values is indicated by *** for *P* < 0.0001. (**F**) Isoforms of the most highly modified gene, *TUG1*. M^6^A modification positions are shown in red. (**G**) Cell type–specific analysis of the genes encoding the top 500 most highly modified isoforms in each brain region.

Of the >50,000 m^6^A modification sites identified in total, there were 5257 and 22,930 identified in all 10 samples or all three brain regions, respectively. Although a majority of modified isoforms were modified in multiple brain regions (67%, *n* = 10,253), 33% were only modified in a single brain region (*n* = 5115) (Fig. 5A). We integrated the results from our DE analysis and found that most of the genes and isoforms modified in only a single brain region were not uniquely expressed or specifically up-regulated in those brain regions (Materials and Methods, Table 2, and table S8). Therefore, region-specific m^6^A modification was not simply due to region-specific expression (*25*).

**Table 2.** Specific m^6^A modification and expression of genes and isoforms in the different brain regions.

	Region-specific m^6^A modification (gene/isoform)	Region-specific DE up-regulation (gene/isoform)	Region-specific m^6^A, no DE up-regulation (gene/isoform)	Region-specific m^6^A, no DE up-regulation % (gene/isoform)
**PFC**	553/1312	1161/2086	449/1065	81%/81%
**CN**	226/508	1593/2930	145/322	64%/63%
**CB**	1413/3295	3227/6184	790/1953	56%/59%

The CB had the largest number of specifically modified features and the largest proportion likely due to increased expression. The PFC exhibited the highest proportion (81%) of modified genes and isoforms not due to expression differences, underscoring the distinctive regional regulation of m^6^A modification in the PFC (Table 2). We performed a GO analysis on isoforms with region-specific m^6^A modification (without region-specific expression) and isoforms commonly modified in all three brain regions. The commonly modified isoforms were primarily associated with protein catabolic terms and, secondarily, neuronal and synaptic terms. CB-specific and CN-specific isoforms had no associations, whereas PFC-specific m^6^A isoforms were associated with multiple synaptic and neuronal cellular components (table S9).

We also performed a cell type–specific enrichment analysis to investigate whether the specifically modified isoforms were associated with different cell types in the different brain regions (*32*). PFC-specific m^6^A isoforms showed the highest degree of enrichment for multiple cell types, including for multiple subtypes of excitatory neurons (Fig. 5B and table S9). The CN-specific isoforms were only enriched for astrocytes, and the CB-specific isoforms were enriched for both cerebellar granule cells (CGCs) and two excitatory neuron subtypes. The integration of our DE analysis with the m^6^A modification data suggests that region-specific modification is spatially regulated by mechanisms other than isoform expression and that there are cell type–specific roles of m^6^A in different brain regions. The cell types associated with specifically modified isoforms are consistent with differences in cell-type composition across brain regions driving isoform-specific modification.

We also found that brain region–specific modification sites had different distributions along an isoform than the common modification sites in all brain regions. The brain region–specific modification sites in PFC and CB had increased densities in the 5′UTR and coding sequence (CDS) and decreased densities in the 3′UTR compared to the common sites (Fig. 5C; Kolmogorov-Smirnov, *P* < 0.0001). The observed divergence in distributions underscores the region-specific regulation of m^6^A modifications.

### Hypermodified and unmodified isoforms

We found that the total number of m^6^A sites per isoform was positively correlated with isoform length (rho = 0.2163, *P* < 0.0001), 3′UTR length (rho = 0.1289, *P* < 0.0001) and negatively correlated with exon density (isoform length/number of exons) (rho = −0.2981, *P* < 0.0001) (*33*, *34*). In agreement with recent studies, unmodified isoforms (*n* = 2339) were generally shorter in length with a higher exon density compared with modified isoforms (MWU, *P* < 0.0001; fig. S6, A and B) (*33*, *34*). We normalized for isoform length and exon density to rank isoforms based on their overall modification levels (Materials and Methods). There was no correlation between the normalized number of m^6^A sites (or raw number of m^6^A sites) and isoform expression (Fig. 5D). However, different transcript isoform biotypes had minor changes in modification levels. Protein-coding and NMD isoforms had higher m^6^A levels than retained intron (RI) and lncRNA isoforms (Fig. 5E).

We found 911 hypermodified isoforms (encoded by 616 genes) (Materials and Methods), and 413 of these isoforms were consistently hypermodified in multiple brain regions (45.33%) (table S10). The top hypermodified isoform in both PFC and CN was from the *PAQR8* gene (ENST00000360726), and in CB was from the *TUG1* lncRNA (ENST00000643071). *PAQR8* was also a top hypermethylated gene in the synaptic compartment of mouse forebrains (*35*). The *TUG1* lncRNA had 37 m^6^A sites, the highest total number observed on a gene in our data (Fig. 5F), and most of these sites were found in all three brain regions. This lncRNA has been associated with glioma stem cell renewal and tumorigenesis, and it has been suggested that lncRNAs may regulate tumor growth through m^6^A modification (*36*, *37*). A study using SCARLET to profile m^6^A in lncRNAs tested 10 sites in *TUG1* for the presence of m^6^A in HeLa, human embryonic kidney–293T, and hepatocellular carcinoma (HEPG2) cell lines (*38*). However, only one site in *TUG1* was m^6^A-modified and identified in all three tested cell lines. A more recent study investigated the m^6^A profile of *TUG1* in two glioma stem cell lines and identified nine m^6^A peaks across the gene (*39*). The variation in m^6^A modification patterns of *TUG1* across different studies and cell lines highlights a need for further research into the regulatory role of m^6^A modification of *TUG1* and other clinically relevant lncRNAs.

The hypermodified isoforms showed enrichment for excitatory neurons in all three brain regions and were highly associated with multiple synapse GO terms and “learning or memory” and “cognition” (fig. S6C and table S11). The PFC hypermodified isoforms were exclusively enriched for excitatory neuron subtypes, whereas the CN and CB isoforms were also enriched for oligodendrocytes and CGCs, respectively (Fig. 5G and table S11). In contrast, unmodified isoforms were associated with cellular metabolism, respiration, and adenosine 5′-triphosphate synthesis GO terms (fig. S6D ansd table S11).

Hypermodified isoforms displayed increased modification density in the CDS compared to all modified isoforms and were not enriched for highly modifiable DRACH motifs (fig. S6, E and F). Previous work identified more CDS m^6^A sites among synaptic FMRP-target RNAs (*25*). The increased density of m^6^A in the CDS of hypermodified RNAs, coupled with their strong enrichment for synaptic processes and consistent association with excitatory neuron cell types, suggests the presence of a unique regulatory environment for synaptic RNA in excitatory neurons and that m^6^A modification has a key role in synaptic function.

### Differences in modification rates of sites between isoforms from the same gene

**Fig. 6.**
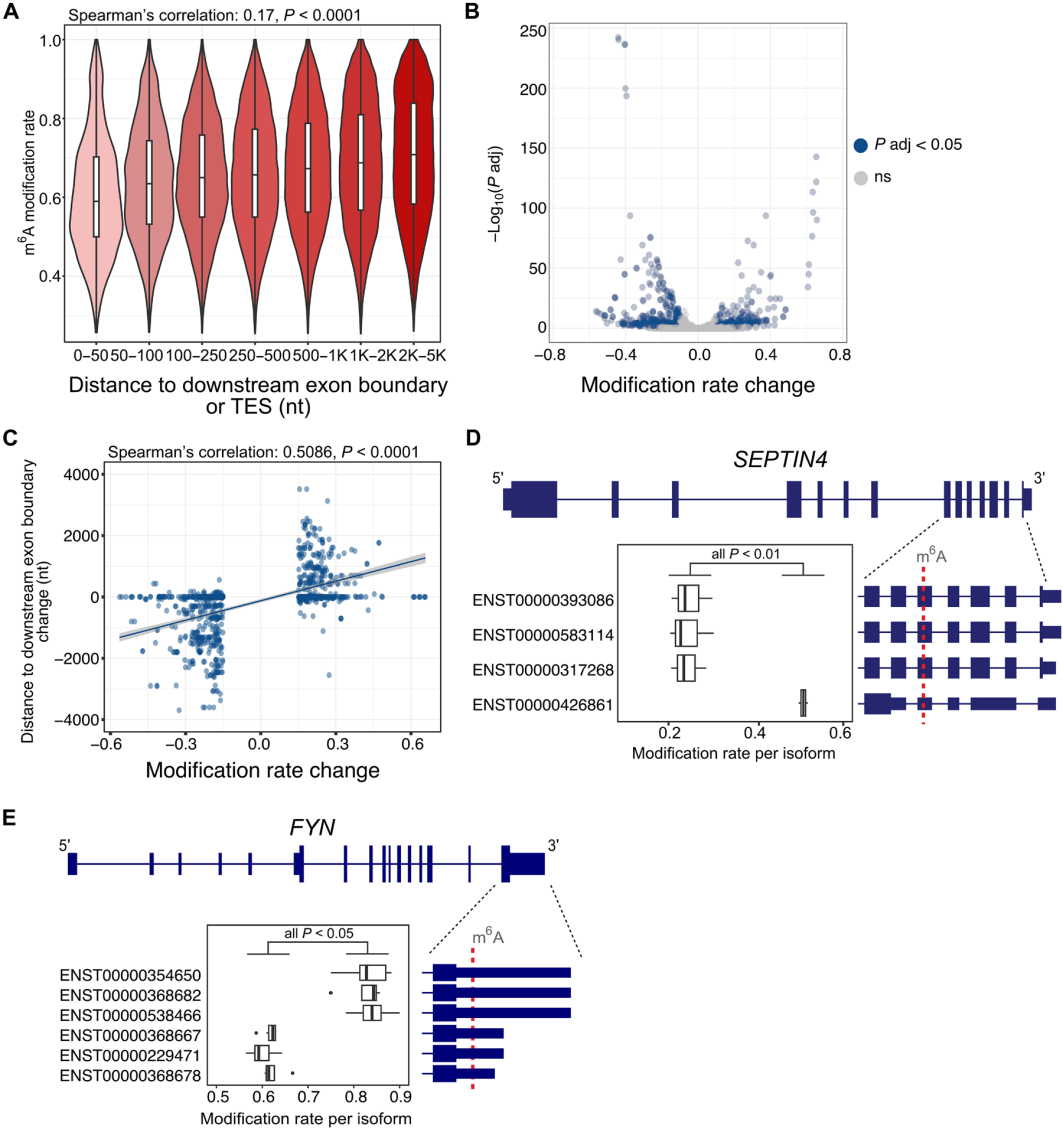
Differences in modification rates of sites between isoforms from the same gene. (**A**) Modification rate of m^6^A sites compared to the distance (in nucleotides, nt) to a downstream exon boundary or transcription end site (TES). (**B**) Volcano plot of modification rate differences within all tested genomic m^6^A sites (*n* = 10,668) and corresponding *p* values adjusted for FDR. Blue indicates significance (FDR < 0.05) in a two-proportion *z* test. (**C**) Modification rate differences of significant sites and corresponding changes in distance to a downstream exon boundary (or TES). (**D**) Genomic m^6^A site within *SEPTIN4* with significant differences in modification rate between isoforms (adjusted *P* value < 0.01). (**E**) Genomic m^6^A site within *FYN* with significant differences in modification rate between isoforms (adjusted *P* value < 0.05). Exact *P* values for each comparison can be found in table S12.

Recent studies have established that the exon junction complex and polyadenylation/transcription termination machinery create an m^6^A modification exclusion zone of ~100 nt on either side of splice junctions and transcription end sites (*33*, *40*). We assessed this in our m^6^A sites and found that distance to a downstream exon boundary was positively correlated with modification rate (rho = 0.1731, *P* < 0.0001) (Fig. 6A). However, the distance to an upstream exon boundary was weakly correlated (rho = 0.0256, *P* < 0.0001).

Building on this, we asked whether isoforms from the same gene could have different modification rates at the same genomic m^6^A site and, if so, whether these differences were associated with changes in isoform structure. We tested 10,668 genomic m^6^A sites encoded in 11,512 isoforms for differences in modification rates, and of these, 828 isoforms had significant differences [two-proportion *z* test, false discovery rate (FDR) < 0.05] at 320 differentially modified (DM) genomic sites (Fig. 6, B and C, and (table S12). We found that an increase in modification rate in an isoform was strongly correlated with an increase in m^6^A site distance to a downstream exon boundary (or transcript end) (rho = 0.5086, *P* < 0.0001) and moderately correlated with an increase in distance from an upstream exon boundary (or transcript start) (rho = 0.2650, *P* < 0.0001). Most of the DM sites (*n* = 264) had a change of >20 nt in the distance to an exon boundary between isoforms, while 30% of the total sites tested (*n* = 3208) had a change of >20 nt in the distance to an exon boundary between isoforms. Therefore, isoform structure is the main driver for isoforms with DM genomic m^6^A sites. However, most genomic sites shared between isoforms are in regions with consistent exonic structures.

Considering that there were DM sites without changes in distances to an exon boundary, we investigated whether the location within the transcript region (5′UTR, CDS, and 3′UTR) could also affect the modification rate. We included this in a linear regression along with the change in distance to an upstream or downstream exon boundary to predict modification rate differences. The model was highly significant [*R*^2^ (coefficient of determination) = 0.4092, *P* < 0.0001] and distances to exon boundaries were the most significant variables (upstream, *P* < 0.01; downstream, *P* < 0.0001). However, we found that genomic sites in 3′UTRs had higher modification rates than the same sites within CDSs (*P* < 0.01). For example, a DM site in *SEPTIN4* in CB had no differences in distance to exon boundaries between isoforms. However, the 3′UTR site still had higher modification rates than the sites within the CDS (Fig. 6D).

There were six isoforms encoding a DM site within *FYN* isoforms that showed the commonly observed pattern of increased distance to a downstream exon boundary and increased modification rate (Fig. 6E). Three isoforms had long 3′UTRs (1407 nt), and three had short 3′UTRs (457 nt). In both PFC and CN, the isoforms with long 3′UTRs had an increase (mean = 0.22) in m^6^A modification rates. Our single-nucleotide isoform-level m^6^A data allow an unbiased view of how mRNA structure affects modification by comparing the same position between different isoforms. Along with finding that regulation of m^6^A deposition can occur in an isoform-specific manner, our differential modification results demonstrate how proximity to splice junctions is not the only cause of differences in modification rates, which are also affected by the distance to transcript ends and the CDS versus UTR status of a nucleotide.

### Differences in modification rates of isoform sites between brain regions

**Fig. 7.**
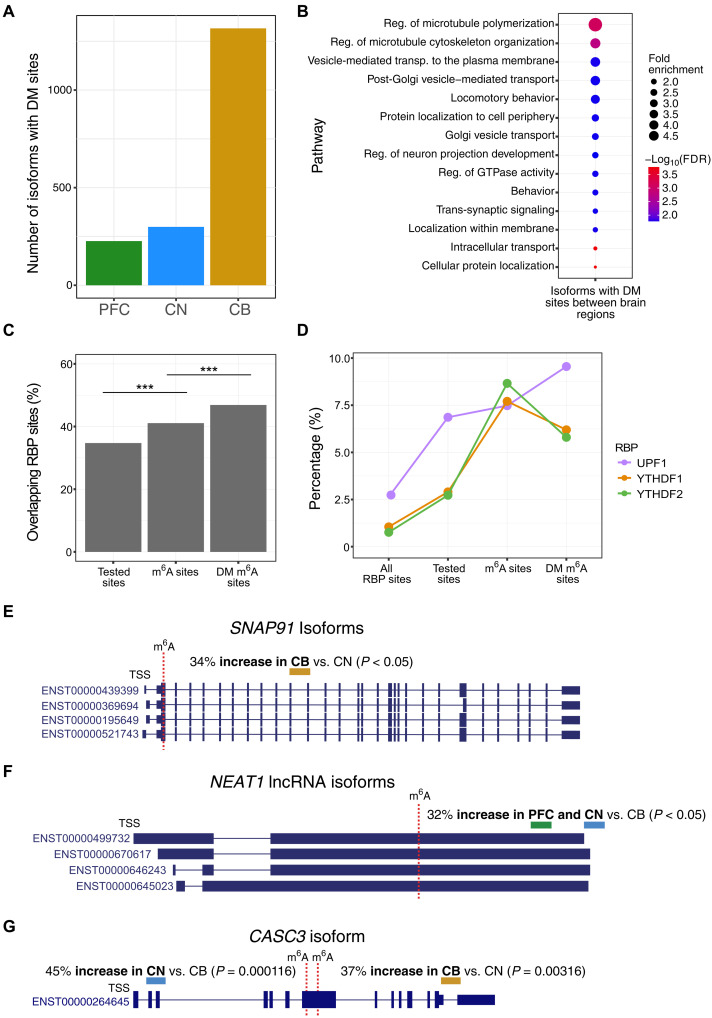
Differences in modification rates within transcriptomic m^6^A sites between brain regions. (**A**) Number of isoforms containing DM m^6^A sites with increased modification rates in each brain region (*n* = 1658). (**B**) GO analysis of all gene isoforms with DM m^6^A sites. (**C**) Percentage of sites per category with genomic coordinates intersecting with RBP coordinates annotated in POSTAR3 and CLIPdb (*43*, *44*). Tested sites are DRACH sites tested for m^6^A modification, m^6^A sites are all m^6^A-modified sites in m6anet, and DM m^6^A sites are DM sites identified by xPore. Two-proportion *z* tests were performed for each comparison and were highly significant (***P < 0.001). (**D**) Percentage of sites in each category that overlap specified RBP binding sites shown only for those with significant increases in either m^6^A sites or DM m^6^A sites. Two-proportion *z* tests for YTHDF1 tested sites versus m^6^A sites, YTHDF2 tested sites versus m^6^A sites, UPF1 m^6^A sites versus DM sites were all significant with *P* < 0.05. (**E**) m^6^A site within four *SNAP91* isoforms with increased modification rates in CB compared to CN (*P* < 0.05). (**F**) m^6^A site within four *NEAT1* lncRNA isoforms with increased modification rates in PFC and CN compared to CB (*P* < 0.05). (**G**) m^6^A site within a *CASC3* isoform with opposing changes in modification rates in an internal exon. The proximal 5′ modification site had increased modification rates in CN compared to CB (*P* < 0.001), whereas the distal 3′ site had increased modification rates (*P* < 0.01). Exact *P* values for each comparison can be found in table S13.

We hypothesized that the same site within an isoform may display brain region–specific differences due to expression differences in m^6^A machinery or cell-type composition between the brain regions. Changes within these sites would not be due to different 3′UTR lengths or proximities to exon boundaries, as the isoform tested is identical between brain regions. We used xPore (*41*) to identify DM transcriptomic sites between brain regions and found 2218 significant DM sites within 1658 isoforms (992 genes) (table S13). Most of the DM sites had an increase in modification rates in CB (*n* = 1666, 75.11%), consistent with the overall levels of m^6^A that we observed in this brain region (Fig. 7A). Isoforms with DM sites were associated with microtubule polymerization, protein transport, and regulation of neuron projection GO terms (Fig. 7B and table S13).

In addition to gene expression changes of the m^6^A machinery in different brain regions, an additional explanation for differential modification of isoforms between brain regions could be due to changes in the activity of different RNA binding proteins (RBPs) at these m^6^A sites. Previous studies have shown that highly m^6^A-modified RNAs interact with more miRNAs and RBPs compared to unmodified RNAs (*42*). We intersected our data with RBP sites annotated in POSTAR3 and CLIPdb (Materials and Methods) (*43*, *44*). We found a notable enrichment for RBP sites in the DM m^6^A sites between brain regions compared with all m^6^A sites and all DRACH sites tested for m^6^A modification (Fig. 7C) (*43*, *45*). The RBPs YTHDF1 and YTHDF2 were significantly enriched at m^6^A sites, while UPF1 was specifically enriched at the DM sites compared with all m^6^A sites (Fig. 7D). UPF1 had significantly increased expression in CB compared with the other brain regions in our gene expression data (*P* < 0.0001) (and in GTEx) and directly interacts with the m^6^A reader protein YTHDF1 to promote rapid degradation of m^6^A-modified RNAs (Fig. 7D) (*2*, *46*). The enrichment of DM m^6^A sites for specific RBPs may highlight a set of isoforms that are spatially regulated between brain regions. The role of UPF1 in promoting RNA degradation may underscore that increased m^6^A levels in CB lead to increased RNA turnover rates, suggesting the importance of this mechanism in this particular brain region. In addition, the enrichment of all m^6^A sites for the m^6^A readers YTHDF1 and YTHDF2 binding sites highlights the accurate identification of m^6^A sites in our dataset.

Four isoforms of *SNAP91* had sites with increased modification rates in CB compared to CN (Fig. 7E). *SNAP91* is involved in synaptic function and is a risk gene for the development of schizophrenia (*47*, *48*). Little information exists about the role of m^6^A modification of *SNAP91*, although it is highly expressed in mouse Purkinje cells, suggesting that this cell type likely drives this observation (*49*). In contrast, a site in *NEAT1* lncRNA isoforms displayed a ~32% increase in m^6^A levels in PFC and CN compared to CB (Fig. 7F). *NEAT1* is essential for the formation of nuclear paraspeckles through extensive interactions with RBPs and is associated with neurodegenerative disorders (*50*, *51*).

Typically, isoforms with DM sites contained only one significant site. However, there were 153 isoforms with multiple DM sites, and the majority of these exhibited a consistent change in direction with other DM sites in the same isoform across the brain regions. For example, two DM sites within the 3′UTR of *NKAIN2* isoforms had a consistent increase in modification rates in CB compared to CN. There were 41 isoforms with inconsistent changes in the direction of modification rates. Two DM sites within a long internal exon (exon 7) of a *CASC3* isoform (ENST00000264645) had opposing changes in modification rates in CN and CB (Fig. 7G). These results indicate that brain region–specific regulation of m^6^A deposition occurs, which does not always follow the general trend observed in our data of increased modification levels in CB, demonstrating how additional factors likely control m^6^A modification at specific isoform sites.

### Regulation of isoform poly(A) lengths between brain regions

Poly(A) tails are critical in posttranscriptional regulation, including in stabilizing mRNA and promoting translation (*52*). We used nanopolish (v0.13.3) to quantify the lengths of poly(A) tails in our human brain samples and tested for global changes in poly(A) tail length between brain regions and isoform-specific changes (*53*).

**Fig. 8.**
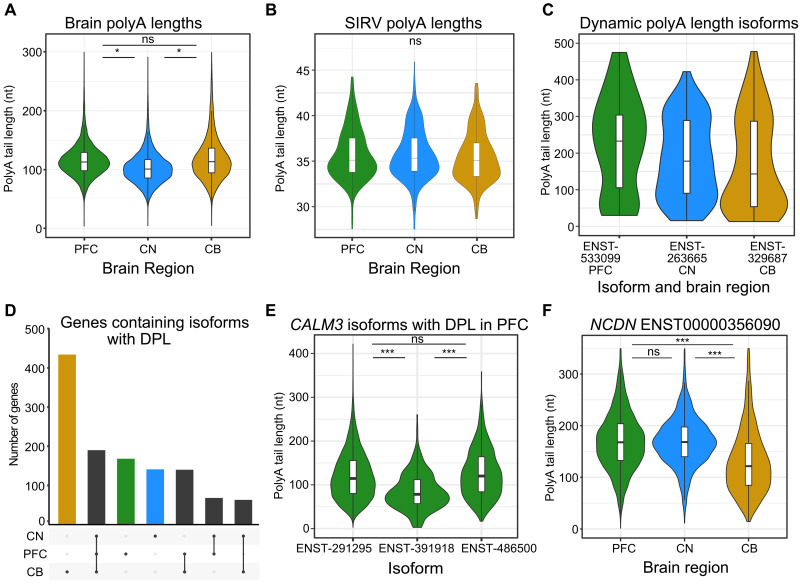
Quantification and changes in polyadenylation lengths between isoforms and brain regions. (**A**) Global poly(A) tail lengths per brain region plotted as a median value for every isoform in each sample. (**B**) Global poly(A) tail lengths of SIRV reads per brain region plotted as a median value for every isoform in each sample. (**C**) Poly(A) tail lengths of the most highly dynamic poly(A) isoform in each brain region. (**D**) UpSet plot of the gene isoforms found with differential polyadenylation lengths (DPLs) within each brain region. (**E**) Poly(A) tail lengths of isoforms with DPL encoded by *CALM3* in PFC plotted for every read. (**F**) Poly(A) tail lengths of an *NCDN* isoform in each brain region were plotted for every read. Significance of *P* values from Mann-Whitney–Wilcoxon tests are indicated by * for <0.05 and *** for <0.001. Ensembl transcript (ENST) IDs omit five 0s for brevity.

Samples from the same brain region mostly clustered together based on the median poly(A) length per isoform, demonstrating consistency between replicates (fig. S7A). Samples from individual 3 were slightly more correlated with each other than samples in their respective brain regions. Globally, CN had shorter poly(A) tail lengths compared with both CB (−16 nt) and PFC (−13 nt) (MWU, *P* < 0.0001) (Fig. 8A). The SIRV data showed no differences in poly(A) lengths between samples, brain regions, or isoforms (Fig. 8B). The poly(A) tails of SIRV isoforms had a median length of 35 nt compared to the ground truth poly(A) length of 30 nt, and poly(A) length estimates per SIRV isoform ranged from 27 to 46 nt.

The median poly(A) length per isoform was positively correlated with annotated isoform length (rho = 0.55, *P* < 0.0001), and we did not observe significant differences in poly(A) lengths between different transcript isoform biotypes (fig. S7B). We noted that some isoforms had considerable variation in their estimated poly(A) lengths, which we termed “dynamic” poly(A) tails (Materials and Methods and table S14). The most dynamic poly(A) length isoforms were from the genes *RNPC3*, *CNTN3*, and *GRIPAP1* in PFC, CN and CB, respectively, and these all had poly(A) tail length ranges of more than 400 nt (Fig. 8C). The genes with dynamic poly(A) lengths were associated with RNA processing GO terms including RNA and ribonucleoprotein export, RNA localization, and RNA splicing (table S15).

We identified 3545 isoforms encoded by 1204 genes with differential poly(A) lengths (DPLs) within genes (table S14). There were 190 genes that had isoforms with DPL in all three brain regions, and a large portion of the genes were exclusively found with DPL in CB (Fig. 8D). We found that isoform length and 3′UTR length were both positively correlated with poly(A) length (rho = 0.5216, *P* < 0.0001 and rho = 0.4146, *P* < 0.0001), and these factors were the main drivers of DPL within genes. For example, isoforms from *CALM3* had DPLs within the PFC (Fig. 8E), and the isoform with the shorter poly(A) lengths (ENST00000391918) also had a short final exon. The genes from isoforms with DPL were associated with translation and splicing GO terms (fig. S7C and table S15).

We next compared poly(A) lengths between the same isoform in different brain regions and identified 566 isoforms with DPLs between brain regions (table S14). These mainly were between isoforms from CN compared to the other brain regions, reflecting the global trend of shorter poly(A) tails in CN. However, this was not always the case, as shown in an isoform from *NCDN* that had longer poly(A) tail lengths in both PFC and CN than CB (Fig. 8F). The isoforms with DPL between brain regions were highly associated with synapse-related and neurotransmitter-related GO terms (fig. S7D and table S15). These results suggest that poly(A) lengths of isoforms are likely under brain region–specific regulation and that this process is particularly important in synaptic function.

### Integrative analysis of isoform expression, poly(A) tail lengths, and m^6^A levels

We investigated whether isoform expression or m^6^A modification levels were related to poly(A) lengths and found a significant overlap (>1.7 times greater than expected by chance) between isoforms with increased poly(A) lengths and up-regulated expression in a brain region (*P* < 0.0001). There was also a significant overlap (>3 times greater than expected by chance) between isoforms from the same gene that had increased modification rates and longer poly(A) lengths within all brain regions (*P* < 0.01). Therefore, when the same gene encodes multiple isoforms, those with distal polyadenylation sites (longer 3′UTRs) will have increased m^6^A modification rates and longer poly(A) tails compared with other isoforms of the same gene. We also found that the number of m^6^A sites on an isoform was moderately correlated with poly(A) tail length (rho = 0.1538, *P* < 0.0001). The observed associations between poly(A) lengths, isoform expression, and m^6^A modification levels highlight the complexity of gene regulation and suggest that the interplay of polyadenylation and m^6^A modification patterns contribute to regulating gene expression in the brain.

### Exploration and visualization of brain m^6^A DRS data

To facilitate online exploration of our DRS datasets and m^6^A results, we have created an online R Shiny App (https://clarklaboratory.shinyapps.io/human_brain_m6a/). Querying a gene of interest will display its isoform-specific expression levels, m^6^A modifications, and poly(A) tail data across each of the three brain regions. To enable visualization of m^6^A sites and modification rates within the context of their genomic positions and the genes and isoforms they are found within, we have enabled a new m^6^A track feature in the Isoform Visualizer (IsoVis) web server and embedded this into the app (fig. S8) (*54*). Researchers can now use IsoVis to view m^6^A data from this or any other study.

## DISCUSSION

Our study is the first to apply DRS on postmortem human brain samples, and we have provided a transcriptome-wide map of >50,000 isoform-level m^6^A modification sites in three distinct brain regions. We identified widespread differences in isoform expression, modification, and polyadenylation profiles between the brain regions. Notably, our findings were consistent across distinct analyses, with expression patterns of m^6^A machinery and isoform architecture reflecting the m^6^A modification levels in the different brain regions.

The highest and lowest proportion of m^6^A modified isoforms and highest and lowest modification rates at m^6^A sites were seen in CB and CN, respectively. Consistent with this finding, m^6^A writers displayed increased expression in CB, and m^6^A erasers had increased expression in CN. We also found that isoform lengths and 3′UTR lengths were longer in isoforms with DE in CB and shorter in isoforms with DE in CN. Together, these results suggest that changes in the expression of the m^6^A machinery and tissue-specific isoform expression patterns are responsible for much of the overall differences in m^6^A levels between the brain regions.

While we found that most m^6^A modified isoforms were commonly modified in multiple brain regions, many were specifically modified in a single brain region, and a majority of these were not due to region-specific up-regulation of isoform expression. Although CB had the largest number of specifically modified isoforms, this region also had the largest proportion that was likely due to increases in expression. The PFC, in contrast, exhibited a distinctly high proportion of specifically modified isoforms that were not due to expression differences, underscoring the unique modification patterns in the PFC. The gene isoforms that displayed region-specific modification were associated with distinct cell types across brain regions. This suggests that region-specific modification may be due to cell type-specific regulation of these isoforms. Isoforms specifically modified in CB were associated with CGCs, and it has been demonstrated that m^6^A is essential in this cell type during cerebellar development (*55*).

We also identified a set of hypermodified isoforms, and many of these were consistently found in all brain regions. Hypermodified isoforms were enriched for excitatory neurons in all three brain regions and highly associated with multiple synapse and postsynapse GO terms, suggesting that the regulation of the m^6^A modification profiles of these isoforms is involved in excitatory neuron function across many brain regions. Our study further emphasizes the region-specific regulatory roles of m^6^A modification within distinct cellular contexts in the brain.

Because of the isoform-level resolution of m^6^A sites in our data, we were able to identify changes in modification rates between the same genomic site encoded in multiple isoforms. Generally, isoforms exhibited similar modification rates at these shared genomic sites, and only ~7% displayed differential modification (DM) rates. Consistent with recent studies, we found that DM between these isoforms was largely attributable to changes in the m^6^A site’s proximity to exon boundaries (*33*, *40*, *56*). We hypothesize that the small degree of DM in our study is a lower bound and increases in read depth, and future improvements to m^6^A detection software will likely enable quantification of modification rates at more sites. Our results suggest that results from methods that provide peaks of m^6^A across gene bodies have likely masked many isoform-specific regulation events of m^6^A deposition.

We also identified thousands of m^6^A-modified isoforms with differences in modification rates (DM) between brain regions, and most of these changes reflected the variable expression levels of m^6^A writers, readers, and erasers. The isoforms with DM were significantly enriched for RBP sites and were also associated with multiple neuronal GO terms and protein transport and microtubule polymerization. Future research into whether these isoform- and tissue-specific m^6^A modifications are a result of distinct cell types or phenotypic states will be an important next step. The identification of m^6^A modifications in single cells is an emerging field and is quickly becoming an area of great interest; however, these methods are still limited by the constraints of SRS (*57*, *58*).

Notably, synapse-related pathways were consistently associated with genes in various analyses in our study. The enrichment of synaptic terms in genes with differential modification and poly(A) lengths between brain regions underscores the relevance of region-specific regulatory processes in synaptic functionality. In addition, the association of synaptic GO terms in genes with DIU between brain regions implies that the expression of specific isoforms is important for modulating synapse activity in a region-dependent manner. Understanding synaptic regulation in various brain regions will be important in uncovering the mechanisms behind many neurological disorders linked to synaptic dysfunction (*59*).

In the future, it will be critical to elucidate the functional role of m^6^A modifications in human neurodegenerative and neuropsychiatric disorders. Exploring how isoform-level m^6^A patterns contribute to diseases such as autism spectrum disorder, schizophrenia, and neurodegenerative conditions will be important for understanding their molecular underpinnings. Furthermore, investigating the evolutionary conservation of m^6^A modification sites and their impact on RNA regulation and function across species may provide insights into their roles in human brain function, development, and disease.

Novel models of human brain development, such as induced pluripotent stem cell-derived cortical and cerebellar organoids, have great potential to provide information on how m^6^A modifications underpin neurodevelopment (*60*). Advancing single cell and isoform-specific m^6^A detection methods will also be paramount for addressing these questions and further refining our understanding of RNA modifications in human health and disease. To this end, the recent release of improved RNA sequencing chemistries and basecalling models from ONT is likely to promote future epitranscriptomics studies, with models now available for the identification of four different RNA modifications (*61*).

A general critique of current m^6^A detection techniques, including immunoprecipitation-based methods, is that the results are not reproducible, and several previous studies have lacked sufficient replicates (*62*). We aimed to address this by including at least three sample replicates per brain region; however, we note the moderate percentage (45%) of m^6^A sites detected in at least three samples per brain region. The number of reads and modification rates at these highly reproducible sites was significantly increased compared to sites with low reproducibility. Consequently, improving the read depth obtained per replicate will likely increase reproducibility at m^6^A sites. However, while recent benchmarking studies have shown that m6anet and xPore perform well, these programs may be limited in their ability to consistently detect m^6^A sites with low modification rates (*63*, *64*). This is observed in our data where very few sites were identified with modifications rates below 40%. Therefore, it is likely that sites with lower stoichiometries are not detected in our dataset, something that newer DRS m^6^A basecalling modules, such as Dorado from ONT, seek to address.

A current limitation of DRS is the relatively large sample input required and low number of reads generated, meaning the technique is not always feasible when only a small amount of sample or tissue is available for RNA isolation. The recent release of updated DRS kits from ONT aims to address the latter of these challenges, and as sequencing throughput for DRS improves, there will be greater power to consistently detect m^6^A sites with low-medium coverage between replicates. Novel basecallers have also recently been introduced that aim to increase read accuracy and improve the single-molecule resolution at m^6^A sites (*65*).

Most gene-level m^6^A sites identified in our data were previously annotated in human tissues in the m^6^A-Atlas or DirectRMDB databases. We noted that m^6^A sites in lncRNAs represented a higher proportion of unannotated sites compared with annotated sites in our data and sites annotated only in m^6^A-Atlas, but not with the DRS-specific resource *DirectRMDB*. This suggests that DRS may be beneficial for investigating m^6^A within lncRNAs compared to previous methods, which will be particularly advantageous for profiling lncRNAs in the human brain, where they have integral roles in learning and memory (*66*, *67*). We also found that the PFC had the highest proportion of both unannotated m^6^A sites and genes found with only unannotated sites of the three brain regions. This result may be due to the additional replicate in PFC (*n* = 4) compared to CB and CN (*n* = 3). However, the total number of m^6^A sites identified was highest in CB, and CN had the second highest proportion of genes with only unannotated sites and the lowest number of m^6^A sites in total. While it is likely that there was increased power to detect more sites in the PFC, it is also possible that genes in this brain region have not been adequately profiled for m^6^A previously.

In summary, our findings have revealed valuable isoform-level insights into three distinct human brain regions. We have demonstrated the interplay of multiple RNA regulatory mechanisms such as isoform expression, m^6^A modification, and polyadenylation. We suggest researchers move toward understanding the functional implications of m^6^A modifications in an isoform-specific and tissue-specific context, and our study supports continued integration of long-read sequencing technologies into the field of RNA modifications.

## MATERIALS AND METHODS

### Sample preparation and quality control

Postmortem brain tissue was obtained from six donors with no diagnosis or physiological evidence of neurological or neuropsychiatric disorders through the Victorian Brain Bank (VBB) under human research ethics committee approval #12457. The age, sex, PMI, brain tissue pH, and brain weight for each individual are shown in table S2. Briefly, samples comprised both males and females (*n* = 3 each), aged between 64 and 81 years, with PMIs between 24 and 59 hours. Frozen brain tissue was cut from three brain regions including PFC (Brodmann’s area 46), CN, and CB. Total RNA was extracted from bulk tissue across five randomized batches. Frozen brain tissue was homogenized on ice using a manual tissue grinder (Potter-Elvehjem, polytetrafluoroethylene) while immersed in 1 ml of QIAzol Lysis Reagent (QIAGEN). The resulting lysate was then made up to 3 ml with QIAzol Lysis Reagent and mixed thoroughly before 1 ml of lysate aliquots were processed using the RNeasy Lipid Tissue Kit 74804 (QIAGEN) according to the manufacturer’s instructions. The increased volume of QIAzol Lysis Reagent was to ensure that each RNA extraction column did not exceed the stated maximum binding capacity of ~100 mg. Three RNA elutions of 30 μl each were combined for a total of 90 μl for each sample. RNA quantity and quality were checked using a Qubit 4 Fluorometer (1 μl), TapeStation 4200, and NanoDrop 2000.

### Library preparation and DRS

Only samples with RNA integrity numbers (RINs) > 7 were selected for long-read DRS, as lower quality RNA was unlikely to yield informative results (*68*). There were 10 high-quality samples for DRS: 4 PFCs, 3 CNs, and 3 CBs. Libraries were prepared on the same day where possible to reduce inter-run variability. Poly(A)^+^ RNA was isolated using NEXTFLEX Poly(A) Beads (PerkinElmer, NOVA-512980) with total RNA inputs ranging from 57 to 100 μg. Isolated poly(A)^+^ RNA (range: 350 to 500 ng) was used for library preparation with the DRS kit SQK-RNA002 (ONT). SIRV Isoform Mix E1 (Lexogen, 025.03) was added to the library at ~1% (~5 ng) of the expected sample poly(A) RNA yield. Prepared libraries were sequenced on the ONT PromethION instrument using FLO-PRO002 flow cells and basecalled with Guppy (v6.0.17) to produce FASTQ files.

### Read alignment and quantification

Pass reads (*q* score > 7) in the FASTQ files were aligned to the human (GRCh38) and SIRV genome and transcriptome using minimap2 (v2.22). Genome alignments were performed using the splice-aware mode of minimap2 *-ax splice -uf -k14*, and transcriptome alignments (GENCODE v31, SIRV) were performed using the long-read mode for ONT data *-ax map-ont*. FeatureCounts (v1.6.5) was used to quantify human and SIRV genome alignments with the parameters *-L –primary* to generate gene counts (*69*). NanoCount was used to quantify human and SIRV transcriptome alignments (v1.1.0) with default parameters to generate isoform counts (*70*). The BamSlam R script was used to obtain summary information regarding the transcriptome alignments outlined in Table 1 (*70*).

### Differential expression and isoform usage analysis

We used limma in R to test for differential gene and isoform expression between the three brain regions (*71*). Log_2_ fold changes and adjusted *P* values (Benjamini-Hochberg) were calculated using the “voomWithQualityWeights” function to account for any variation in sample quality and adjusted *P* values < 0.05 were required for significance (*72*). DIU analysis was performed in R using IsoformSwitchAnalyzeR (*73*). The isoform counts from NanoCount were input along with the annotation and transcriptome files. Statistical analysis was performed with DEXSeq to identify differential isoform usage between brain regions (*74*). The count matrix was filtered for genes with >1 isoform, genes with >20 counts, and isoforms with >5 counts. We required a change in isoform proportions between brain regions of >0.2 to further increase stringency and an FDR-adjusted *P* value of <0.05 for significance (*75*).

### Transcriptome-wide m^6^A modification sites

We used m6anet (v2.0.1) to identify m^6^A sites in DRACH motifs (D: A, G, or U, R: A or G, and H: A, C, or U) from our direct RNA reads in each sample. The program outputs an m^6^A modification probability and modification rate (proportion of modified reads) at every transcript isoform site with a coverage of >20 reads (*18*). There were ~30 million reads mapped to sites meeting the read coverage requirements that were tested for m^6^A modifications. We required a modification probability of >0.90 in >1 sample for the site to be classed as m^6^A modified (*n* = 57,144 unique m^6^A sites and *n* = 228,314 total m^6^A sites across all samples).

### Identification of common and brain region–specific m^6^A isoform modifications

Starting from our 57,144 high-confidence m^6^A sites, genes and isoforms that had at least one m^6^A modification site that was identified in all three brain regions were termed commonly modified. To identify brain region–specific modification, we subset genes and isoforms to only retain those with m^6^A modification(s) in a single brain region. We further distinguished between genes and isoforms that were identified as modified in a specific brain region due to expression up-regulation versus region-specific modification using our differential expression results. Genes and isoforms modified only in the region where they were significantly up-regulated were considered to be identified due to expression changes. Removing these gave the nonexpression-related region-specific modified genes and isoforms. The common and nonexpression-related brain region–specific m^6^A modified genes were used in a GO and cell type–specific enrichment analysis. The distributions of common or brain region–specific m^6^A modifications along isoforms were compared using a Kolgomorov-Smirnov test (Fig. 5C).

### Comparison with annotated m^6^A sites in m^6^A-Atlas and DirectRMDB

We downloaded data from m^6^A-Atlas (v2.0) and DirectRMDB to identify genomic m^6^A sites in our data that were previously annotated (*30*, *31*). The data were subset for only human cell lines or tissues for comparison.

### Hypermodified and unmodified isoforms

We calculated a normalized number of m^6^A modifications per isoform by summing the modification rates at every m^6^A site along the isoform and using this along with isoform length and exon density in a linear regression as predictor variables. We extracted the residuals from this model and used these as normalized m^6^A values. We defined the hypermodified isoforms as the top 500 per brain region ranked by normalized m^6^A values. The unmodified isoforms were defined as those with no m^6^A modifications that also had adequate coverage for m^6^A detection (>1 DRACH motif detected in m6anet) and a modification probability of <0.5 at all detected sites within the isoform.

### Identification of differential modification between isoforms and brain regions

We used a two-proportion *z* test to identify differential modification (DM) rates between isoforms encoding the same genomic m^6^A site within a brain region. We required an FDR-adjusted *P* value <0.05 and a modification rate difference between isoforms of >0.15 for significance.

To test for differential modification at the same site in an isoform between different brain regions, we integrated the results from m6anet and xPore (*41*). xPore (v2.1) identifies sites with differential modification rates between conditions but does not identify the type of modification present when it is run without an unmodified control sample. We subset the xPore sites for DRACH motifs, a modification rate difference of >0.3 and an FDR-adjusted *P* value < 0.05 as recommended (*41*). These sites were then overlapped with sites found in m6anet (modification probability score > 0.7) in >1 sample to create the final list of DM sites.

### Poly(A) tail length quantification and analysis

The poly(A) tail lengths for each read were estimated using the “polya” module from nanopolish (v0.13.2) (*53*). We kept reads assigned a “pass” QC tag from nanopolish and required >5 reads per isoform per sample. Consistent with previous studies, we found that mitochondrial isoforms had shorter poly(A) tail lengths than nonmitochondrial isoforms, so these were excluded from downstream analysis (*53*). We calculated a median poly(A) tail length for each isoform in each sample to avoid highly expressed isoforms skewing the comparisons. We performed a MWU test to compare the overall median poly(A) lengths between the brain regions and between isoforms of the same gene. We used a MWU test for this comparison and required >50 reads and a poly(A) length difference of >20 nt per isoform.

We noted that some isoforms had large variations in their estimated poly(A) lengths, termed dynamic poly(A) tails. We used the interquartile range (IQR) of isoforms with >50 reads per brain region to rank the top 250 isoforms according to variations in poly(A) lengths per brain region. IQR was used to prevent a small number of outliers from influencing the ranking.

### Statistical analysis and visualization

We used R for all statistical analysis and plotting unless otherwise stated. The MWU test was used for statistical comparisons with the wilcox.test() function from the “stats” package, and *P* values were subsequently FDR-adjusted when multiple comparisons were performed with the p.adjust() function also from the stats package. Linear regressions were performed using the lm() function, and their respective summaries were extracted using the summary() function. The metagene plots were produced using metaPlotR (*76*). Hypergeometric tests were performed with phyper to obtain *P* values for the number of overlapping isoforms between different analyses.

We used the R package clusterProfiler (v4.6.2) to perform the various GO analyses in this study (*77*). The enrichGO() function was applied to gene sets using relevant background genes (i.e., expressed genes and m^6^A-modified genes). All three GO domains were included: biological process, molecular function, and cellular compartment. *P* values were adjusted for the FDR (adjusted *P* value < 0.05), and redundant GO terms were removed using the simplify function with a cutoff value of 0.7. We removed GO terms with <10 genes assigned to the pathway and also calculated an enrichment value for each GO term as per ShinyGO (v0.77) defined as the percentage of genes belonging to a pathway divided by the corresponding percentage of background genes belonging to the pathway (*78*).

To perform the cell type–specific enrichment analysis we used WebCSEA (*32*) and required a combined *P* value < 0.001 for significance and subset the results for the “adult” development stage and “nervous system” organ system as recommended.

Data for all annotated human RBP sites were downloaded from POSTAR3 and CLIPdb (*43*, *44*). The sites were filtered for those annotated at least two times. We used bedtools to intersect the RBP sites with genomic coordinates of all DRACH sites tested with m6anet, all m^6^A sites identified by m6anet, and m^6^A sites with differential modification rates between brain regions identified by xPore, with the following command: bedtools window -a xPore_genomic_positions.bed -b human_RBP_sites.bed -w 5 -u > result.bed. We used the two-proportion *z* test to determine whether there were significant differences in proportions of sites overlapping RBP sites (Fig. 7, C and D).

We developed an R Shiny application to explore and visualize the data from our study. The “Explore data” tab allows users to query a gene and displays tables of the expression, m^6^A modification and poly(A) information per isoform per brain region. The “IsoVis” tab provides files for download and an embedded IsoVis webserver to visualize m^6^A genomic positions and modification rates (*54*). This is publicly available at https://clarklaboratory.shinyapps.io/human_brain_m6a/.

## References

[1] T. W. Nilsen, B. R. Graveley, Expansion of the eukaryotic proteome by alternative splicing. Nature 463, 457–463 (2010). 20110989 10.1038/nature08909 PMC3443858

[2] GTEx Consortium, The genotype-tissue expression (GTEx) pilot analysis: Multitissue gene regulation in humans. Science 348, 648–660 (2015). 25954001 10.1126/science.1262110 PMC4547484

[3] B. Raj, B. J. Blencowe, Alternative splicing in the mammalian nervous system: Recent insights into mechanisms and functional roles. Neuron 87, 14–27 (2015). 26139367 10.1016/j.neuron.2015.05.004

[4] C.-H. Su, D. Dhananjaya, W.-Y. Tarn, Alternative splicing in neurogenesis and brain development. Front. Mol. Biosci. 5, 12 (2018). 29484299 10.3389/fmolb.2018.00012 PMC5816070

[5] K. D. Meyer, Y. Saletore, P. Zumbo, O. Elemento, C. E. Mason, S. R. Jaffrey, Comprehensive analysis of mRNA methylation reveals enrichment in 3′ UTRs and near stop codons. Cell 149, 1635–1646 (2012). 22608085 10.1016/j.cell.2012.05.003 PMC3383396

[6] D. Dominissini, S. Moshitch-Moshkovitz, S. Schwartz, M. Salmon-Divon, L. Ungar, S. Osenberg, K. Cesarkas, J. Jacob-Hirsch, N. Amariglio, M. Kupiec, R. Sorek, G. Rechavi, Topology of the human and mouse m6A RNA methylomes revealed by m6A-seq. Nature 485, 201–206 (2012). 22575960 10.1038/nature11112

[7] I. Livneh, S. Moshitch-Moshkovitz, N. Amariglio, G. Rechavi, D. Dominissini, The m^6^A epitranscriptome: Transcriptome plasticity in brain development and function. Nat. Rev. Neurosci. 21, 36–51 (2020). 31804615 10.1038/s41583-019-0244-z

[8] A. M. Shafik, E. G. Allen, P. Jin, Dynamic N^6^-methyladenosine RNA methylation in brain and diseases. Epigenomics 12, 371–380 (2020). 32081027 10.2217/epi-2019-0260 PMC7132785

[9] J. Liu, K. Li, J. Cai, M. Zhang, X. Zhang, X. Xiong, H. Meng, X. Xu, Z. Huang, J. Peng, J. Fan, C. Yi, Landscape and regulation of m^6^A and m^6^Am methylome across human and mouse tissues. Mol. Cell 77, 426–440.e6 (2020). 31676230 10.1016/j.molcel.2019.09.032

[10] J. Mathoux, D. C. Henshall, G. P. Brennan, Regulatory mechanisms of the RNA modification m^6^A and significance in brain function in health and disease. Front. Cell. Neurosci. 15, 671932 (2021). 34093133 10.3389/fncel.2021.671932 PMC8170084

[11] J. Widagdo, V. Anggono, The m^6^A-epitranscriptomic signature in neurobiology: From neurodevelopment to brain plasticity. J. Neurochem. 147, 137–152 (2018). 29873074 10.1111/jnc.14481

[12] S. U. Madugalle, K. Meyer, D. O. Wang, T. W. Bredy, RNA N^6^-methyladenosine and the regulation of RNA localization and function in the brain. Trends Neurosci. 43, 1011–1023 (2020). 33041062 10.1016/j.tins.2020.09.005 PMC7688512

[13] B. Molinie, C. C. Giallourakis, RNA methylation, methods and protocols. Methods Mol. Biol. 1562, 45–53 (2017). 28349453 10.1007/978-1-4939-6807-7_4 PMC5755608

[14] H. Zheng, X. Zhang, N. Sui, Advances in the profiling of N^6^-methyladenosine (m^6^A) modifications. Biotechnol. Adv. 45, 107656 (2020). 33181242 10.1016/j.biotechadv.2020.107656

[15] Y.-L. Xiao, S. Liu, R. Ge, Y. Wu, C. He, M. Chen, W. Tang, Transcriptome-wide profiling and quantification of N^6^-methyladenosine by enzyme-assisted adenosine deamination. Nat. Biotechnol. 41, 993–1003 (2023). 36593412 10.1038/s41587-022-01587-6 PMC10625715

[16] C. Liu, H. Sun, Y. Yi, W. Shen, K. Li, Y. Xiao, F. Li, Y. Li, Y. Hou, B. Lu, W. Liu, H. Meng, J. Peng, C. Yi, J. Wang, Absolute quantification of single-base m^6^A methylation in the mammalian transcriptome using GLORI. Nat. Biotechnol. 41, 355–366 (2023). 36302990 10.1038/s41587-022-01487-9

[17] P. A. Mateos, A. J. Sethi, A. Ravindran, A. Srivastava, K. Woodward, S. Mahmud, M. Kanchi, M. Guarnacci, J. Xu, Z. W. S. Yuen, Y. Zhou, A. Sneddon, W. Hamilton, J. Gao, L. M. Starrs, R. Hayashi, V. Wickramasinghe, K. Zarnack, T. Preiss, G. Burgio, N. Dehorter, N. E. Shirokikh, E. Eyras, Prediction of m^6^A and m^5^C at single-molecule resolution reveals a transcriptome-wide co-occurrence of RNA modifications. Nat. Commun. 15, 3899 (2024). 38724548 10.1038/s41467-024-47953-7 PMC11082244

[18] C. Hendra, P. N. Pratanwanich, Y. K. Wan, W. S. S. Goh, A. Thiery, J. Göke, Detection of m^6^A from direct RNA sequencing using a multiple instance learning framework. Nat. Methods 19, 1590–1598 (2022). 36357692 10.1038/s41592-022-01666-1 PMC9718678

[19] E. Sjöstedt, W. Zhong, L. Fagerberg, M. Karlsson, N. Mitsios, C. Adori, P. Oksvold, F. Edfors, A. Limiszewska, F. Hikmet, J. Huang, Y. Du, L. Lin, Z. Dong, L. Yang, X. Liu, H. Jiang, X. Xu, J. Wang, H. Yang, L. Bolund, A. Mardinoglu, C. Zhang, K. von Feilitzen, C. Lindskog, F. Pontén, Y. Luo, T. Hökfelt, M. Uhlén, J. Mulder, An atlas of the protein-coding genes in the human, pig, and mouse brain. Science 367, 6482 (2020). 10.1126/science.aay5947 32139519

[20] Schizophrenia Working Group of the Psychiatric Genomics Consortium, Biological insights from 108 schizophrenia-associated genetic loci. Nature 511, 421–427 (2014). 25056061 10.1038/nature13595 PMC4112379

[21] Schizophrenia Working Group of the Psychiatric Genomics Consortium, Mapping genomic loci implicates genes and synaptic biology in schizophrenia. Nature 604, 502–508 (2022). 35396580 10.1038/s41586-022-04434-5 PMC9392466

[22] M. Uhlén, L. Fagerberg, B. M. Hallström, C. Lindskog, P. Oksvold, A. Mardinoglu, Å. Sivertsson, C. Kampf, E. Sjöstedt, A. Asplund, I. Olsson, K. Edlund, E. Lundberg, S. Navani, C. A.-K. Szigyarto, J. Odeberg, D. Djureinovic, J. O. Takanen, S. Hober, T. Alm, P.-H. Edqvist, H. Berling, H. Tegel, J. Mulder, J. Rockberg, P. Nilsson, J. M. Schwenk, M. Hamsten, K. von Feilitzen, M. Forsberg, L. Persson, F. Johansson, M. Zwahlen, G. von Heijne, J. Nielsen, F. Pontén, Tissue-based map of the human proteome. Science 347, 1260419 (2015). 25613900 10.1126/science.1260419

[23] K. Horiuchi, T. Kawamura, H. Iwanari, R. Ohashi, M. Naito, T. Kodama, T. Hamakubo, Identification of Wilms’ tumor 1-associating protein complex and its role in alternative splicing and the cell cycle. J. Biol. Chem. 288, 33292–33302 (2013). 24100041 10.1074/jbc.M113.500397 PMC3829175

[24] X. Yan, K. Pei, Z. Guan, F. Liu, J. Yan, X. Jin, Q. Wang, M. Hou, C. Tang, P. Yin, AI-empowered integrative structural characterization of m^6^A methyltransferase complex. Cell Res. 32, 1124–1127 (2022). 36357785 10.1038/s41422-022-00741-8 PMC9715571

[25] M. Chang, H. Lv, W. Zhang, C. Ma, X. He, S. Zhao, Z.-W. Zhang, Y.-X. Zeng, S. Song, Y. Niu, W.-M. Tong, Region-specific RNA m^6^A methylation represents a new layer of control in the gene regulatory network in the mouse brain. Open Biol. 7, 170166 (2017). 28931651 10.1098/rsob.170166 PMC5627058

[26] C. Ma, M. Chang, H. Lv, Z.-W. Zhang, W. Zhang, X. He, G. Wu, S. Zhao, Y. Zhang, D. Wang, X. Teng, C. Liu, Q. Li, A. Klungland, Y. Niu, S. Song, W.-M. Tong, RNA m^6^A methylation participates in regulation of postnatal development of the mouse cerebellum. Genome Biol. 19, 68 (2018). 29855379 10.1186/s13059-018-1435-z PMC5984455

[27] S. Ke, E. A. Alemu, C. Mertens, E. C. Gantman, J. J. Fak, A. Mele, B. Haripal, I. Zucker-Scharff, M. J. Moore, C. Y. Park, C. B. Vågbø, A. Kusśnierczyk, A. Klungland, J. E. Darnell, R. B. Darnell, A majority of m^6^A residues are in the last exons, allowing the potential for 3′ UTR regulation. Genes Dev. 29, 2037–2053 (2015). 26404942 10.1101/gad.269415.115 PMC4604345

[28] M. T. Parker, K. Knop, A. V. Sherwood, N. J. Schurch, K. Mackinnon, P. D. Gould, A. J. Hall, G. J. Barton, G. G. Simpson, Nanopore direct RNA sequencing maps the complexity of Arabidopsis mRNA processing and m^6^A modification. eLife 9, e49658 (2020). 31931956 10.7554/eLife.49658 PMC6959997

[29] B. Linder, A. V. Grozhik, A. O. Olarerin-George, C. Meydan, C. E. Mason, S. R. Jaffrey, Single-nucleotide-resolution mapping of m^6^A and m^6^Am throughout the transcriptome. Nat. Methods 12, 767–772 (2015). 26121403 10.1038/nmeth.3453 PMC4487409

[30] Y. Zhang, J. Jiang, J. Ma, Z. Wei, Y. Wang, B. Song, J. Meng, G. Jia, J. P. de Magalhães, D. J. Rigden, D. Hang, K. Chen, DirectRMDB: A database of post-transcriptional RNA modifications unveiled from direct RNA sequencing technology. Nucleic Acids Res. 51, D106–D116 (2022). 10.1093/nar/gkac1061 PMC9825532 36382409

[31] Z. Liang, H. Ye, J. Ma, Z. Wei, Y. Wang, Y. Zhang, D. Huang, B. Song, J. Meng, D. J. Rigden, m^6^A-Atlas v2.0: Updated resources for unraveling the N6-methyladenosine (m^6^A) epitranscriptome among multiple species. Nucleic Acids Res. 52, D194–D202 (2024). 37587690 10.1093/nar/gkad691 PMC10768109

[32] Y. Dai, R. Hu, A. Liu, K. S. Cho, A. M. Manuel, X. Li, X. Dong, P. Jia, Z. Zhao, WebCSEA: Web-based cell-type-specific enrichment analysis of genes. Nucleic Acids Res. 50, W782–W790 (2022). 35610053 10.1093/nar/gkac392 PMC10359109

[33] A. Uzonyi, D. Dierks, R. Nir, O. S. Kwon, U. Toth, I. Barbosa, C. Burel, A. Brandis, W. Rossmanith, H. L. Hir, B. Slobodin, S. Schwartz, Exclusion of m^6^A from splice-site proximal regions by the exon junction complex dictates m^6^A topologies and mRNA stability. Mol. Cell 83, 237–251.e7 (2023). 36599352 10.1016/j.molcel.2022.12.026

[34] X. Yang, R. Triboulet, Q. Liu, E. Sendinc, R. I. Gregory, Exon junction complex shapes the m^6^A epitranscriptome. Nat. Commun. 13, 7904 (2022). 36550132 10.1038/s41467-022-35643-1 PMC9780246

[35] D. Merkurjev, W.-T. Hong, K. Iida, I. Oomoto, B. J. Goldie, H. Yamaguti, T. Ohara, S. Kawaguchi, T. Hirano, K. C. Martin, M. Pellegrini, D. O. Wang, Synaptic *N*^6^-methyladenosine (m^6^A) epitranscriptome reveals functional partitioning of localized transcripts. Nat. Neurosci. 21, 1004–1014 (2018). 29950670 10.1038/s41593-018-0173-6

[36] K. Katsushima, A. Natsume, F. Ohka, K. Shinjo, A. Hatanaka, N. Ichimura, S. Sato, S. Takahashi, H. Kimura, Y. Totoki, T. Shibata, M. Naito, H. J. Kim, K. Miyata, K. Kataoka, Y. Kondo, Targeting the Notch-regulated non-coding RNA TUG1 for glioma treatment. Nat. Commun. 7, 13616 (2016). 27922002 10.1038/ncomms13616 PMC5150648

[37] H. Cai, X. Liu, J. Zheng, Y. Xue, J. Ma, Z. Li, Z. Xi, Z. Li, M. Bao, Y. Liu, Long non-coding RNA taurine upregulated 1 enhances tumor-induced angiogenesis through inhibiting microRNA-299 in human glioblastoma. Oncogene 36, 318–331 (2017). 27345398 10.1038/onc.2016.212

[38] N. Liu, M. Parisien, Q. Dai, G. Zheng, C. He, T. Pan, Probing N^6^-methyladenosine RNA modification status at single nucleotide resolution in mRNA and long noncoding RNA. RNA 19, 1848–1856 (2013). 24141618 10.1261/rna.041178.113 PMC3884656

[39] G. Steponaitis, R. Stakaitis, I. Valiulyte, R. Krusnauskas, R. Dragunaite, R. Urbanavičiūtė, A. Tamasauskas, D. Skiriute, Transcriptome-wide analysis of glioma stem cell specific m^6^A modifications in long-non-coding RNAs. Sci. Rep. 12, 5431 (2022). 35361860 10.1038/s41598-022-08616-z PMC8971438

[40] P. C. He, J. Wei, X. Dou, B. T. Harada, Z. Zhang, R. Ge, C. Liu, L.-S. Zhang, X. Yu, S. Wang, R. Lyu, Z. Zou, M. Chen, C. He, Exon architecture controls mRNA m^6^A suppression and gene expression. Science 379, 677–682 (2023). 36705538 10.1126/science.abj9090 PMC9990141

[41] P. N. Pratanwanich, F. Yao, Y. Chen, C. W. Q. Koh, Y. K. Wan, C. Hendra, P. Poon, Y. T. Goh, P. M. L. Yap, J. Y. Chooi, W. J. Chng, S. B. Ng, A. Thiery, W. S. S. Goh, J. Göke, Identification of differential RNA modifications from nanopore direct RNA sequencing with xPore. Nat. Biotechnol. 39, 1394–1402 (2021). 34282325 10.1038/s41587-021-00949-w

[42] S. D. Mandal, P. S. Ray, Transcriptome-wide analysis reveals spatial correlation between N^6^-methyladenosine and binding sites of microRNAs and RNA-binding proteins. Genomics 113, 205–216 (2021). 33340693 10.1016/j.ygeno.2020.12.027

[43] Y.-C. T. Yang, C. Di, B. Hu, M. Zhou, Y. Liu, N. Song, Y. Li, J. Umetsu, Z. J. Lu, CLIPdb: A CLIP-seq database for protein-RNA interactions. BMC Genomics 16, 51 (2015). 25652745 10.1186/s12864-015-1273-2 PMC4326514

[44] W. Zhao, S. Zhang, Y. Zhu, X. Xi, P. Bao, Z. Ma, T. H. Kapral, S. Chen, B. Zagrovic, Y. T. Yang, Z. J. Lu, POSTAR3: An updated platform for exploring post-transcriptional regulation coordinated by RNA-binding proteins. Nucleic Acids Res. 50, D287–D294 (2022). 34403477 10.1093/nar/gkab702 PMC8728292

[45] W. Hong, Y. Zhao, Y.-L. Weng, C. Cheng, Random Forest model reveals the interaction between N^6^-methyladenosine modifications and RNA-binding proteins. iScience 26, 106250 (2023). 36922995 10.1016/j.isci.2023.106250 PMC10009289

[46] S. H. Boo, H. Ha, Y. Lee, M.-K. Shin, S. Lee, Y. K. Kim, UPF1 promotes rapid degradation of m^6^A-containing RNAs. Cell Rep. 39, 110861 (2022). 35613594 10.1016/j.celrep.2022.110861

[47] S. Nadine, H. Man Seok, Y. Kazuhiko, D. Amanda, H. Laura, M. R. Marliette, C. Esther, P. J. Michael Deans, F. Erin, B. Natalie, T. Aaron, A. Khaled, A. Sonya, G. James, H. Emily, P. Hemali, S. Vineeta, G. Deeptha, A. Bruce, M. Robert, H. E. Gabriel, S. A. Eli, M. Hirofumi, S. Pamela, B. J. Kristen, Synergistic effects of common schizophrenia risk variants. Nat. Genet. 51, 1475–1485 (2019). 31548722 10.1038/s41588-019-0497-5 PMC6778520

[48] E. S. Lips, L. N. Cornelisse, R. F. Toonen, J. L. Min, C. M. Hultman, I. S. Consortium, P. A. Holmans, M. C. O’Donovan, S. M. Purcell, A. B. Smit, M. Verhage, P. F. Sullivan, P. M. Visscher, D. Posthuma, Functional gene group analysis identifies synaptic gene groups as risk factor for schizophrenia. Mol. Psychiatry 17, 996–1006 (2012). 21931320 10.1038/mp.2011.117 PMC3449234

[49] V. Kozareva, C. Martin, T. Osorno, S. Rudolph, C. Guo, C. Vanderburg, N. Nadaf, A. Regev, W. G. Regehr, E. Macosko, A transcriptomic atlas of mouse cerebellar cortex comprehensively defines cell types. Nature 598, 214–219 (2021). 34616064 10.1038/s41586-021-03220-z PMC8494635

[50] H. An, N. G. Williams, T. A. Shelkovnikova, NEAT1 and paraspeckles in neurodegenerative diseases: A missing lnc found? Non-Coding RNA Res. 3, 243–252 (2018). 10.1016/j.ncrna.2018.11.003 PMC6257911 30533572

[51] C. M. Clemson, J. N. Hutchinson, S. A. Sara, A. W. Ensminger, A. H. Fox, A. Chess, J. B. Lawrence, An architectural role for a nuclear noncoding RNA: NEAT1 RNA is essential for the structure of paraspeckles. Mol. Cell 33, 717–726 (2009). 19217333 10.1016/j.molcel.2009.01.026 PMC2696186

[52] L. A. Passmore, J. Coller, Roles of mRNA poly(A) tails in regulation of eukaryotic gene expression. Nat. Rev. Mol. Cell Biol. 23, 93–106 (2022). 34594027 10.1038/s41580-021-00417-y PMC7614307

[53] R. E. Workman, A. D. Tang, P. S. Tang, M. Jain, J. R. Tyson, R. Razaghi, P. C. Zuzarte, T. Gilpatrick, A. Payne, J. Quick, N. Sadowski, N. Holmes, J. G. de Jesus, K. L. Jones, C. M. Soulette, T. P. Snutch, N. Loman, B. Paten, M. Loose, J. T. Simpson, H. E. Olsen, A. N. Brooks, M. Akeson, W. Timp, Nanopore native RNA sequencing of a human poly(A) transcriptome. Nat. Methods 16, 1297–1305 (2019). 31740818 10.1038/s41592-019-0617-2 PMC7768885

[54] C. Y. Wan, J. Davis, M. Chauhan, J. Gleeson, Y. D. J. Prawer, R. D. Paoli-Iseppi, C. A. Wells, J. Choi, M. B. Clark, IsoVis – A webserver for visualization and annotation of alternative RNA isoforms. Nucleic Acids Res. 52, W341–W347 (2024). 38709877 10.1093/nar/gkae343 PMC11223830

[55] C.-X. Wang, G.-S. Cui, X. Liu, K. Xu, M. Wang, X.-X. Zhang, L.-Y. Jiang, A. Li, Y. Yang, W.-Y. Lai, B.-F. Sun, G.-B. Jiang, H.-L. Wang, W.-M. Tong, W. Li, X.-J. Wang, Y.-G. Yang, Q. Zhou, METTL3-mediated m6A modification is required for cerebellar development. PLOS Biol. 16, e2004880 (2018). 29879109 10.1371/journal.pbio.2004880 PMC6021109

[56] Z. Luo, Q. Ma, S. Sun, N. Li, H. Wang, Z. Ying, S. Ke, Exon-intron boundary inhibits m^6^A deposition, enabling m^6^A distribution hallmark, longer mRNA half-life and flexible protein coding. Nat. Commun. 14, 4172 (2023). 37443320 10.1038/s41467-023-39897-1 PMC10345190

[57] M. Tegowski, M. N. Flamand, K. D. Meyer, scDART-seq reveals distinct m^6^A signatures and mRNA methylation heterogeneity in single cells. Mol. Cell 82, 868–878.e10 (2022). 35081365 10.1016/j.molcel.2021.12.038 PMC8857065

[58] H. Yao, C.-C. Gao, D. Zhang, J. Xu, G. Song, X. Fan, D.-B. Liang, Y.-S. Chen, Q. Li, Y. Guo, Y.-T. Cai, L. Hu, Y.-L. Zhao, Y.-P. Sun, Y. Yang, J. Han, Y.-G. Yang, scm6A-seq reveals single-cell landscapes of the dynamic m^6^A during oocyte maturation and early embryonic development. Nat. Commun. 14, 315 (2023). 36658155 10.1038/s41467-023-35958-7 PMC9852475

[59] E. Taoufik, G. Kouroupi, O. Zygogianni, R. Matsas, Synaptic dysfunction in neurodegenerative and neurodevelopmental diseases: An overview of induced pluripotent stem-cell-based disease models. Open Biol. 8, 180138 (2018). 30185603 10.1098/rsob.180138 PMC6170506

[60] S. Velasco, A. J. Kedaigle, S. K. Simmons, A. Nash, M. Rocha, G. Quadrato, B. Paulsen, L. Nguyen, X. Adiconis, A. Regev, J. Z. Levin, P. Arlotta, Individual brain organoids reproducibly form cell diversity of the human cerebral cortex. Nature 570, 523–527 (2019). 31168097 10.1038/s41586-019-1289-x PMC6906116

[61] Oxford Nanopore Technologies, Dorado basecaller. [Software tool] (2024). https://github.com/nanoporetech/dorado.

[62] A. B. R. McIntyre, N. S. Gokhale, L. Cerchietti, S. R. Jaffrey, S. M. Horner, C. E. Mason, Limits in the detection of m^6^A changes using MeRIP/m^6^A-seq. Sci. Rep. 10, 6590 (2020). 32313079 10.1038/s41598-020-63355-3 PMC7170965

[63] S. Maestri, M. Furlan, L. Mulroney, L. C. Tarrero, C. Ugolini, F. D. Pozza, T. Leonardi, E. Birney, F. Nicassio, M. Pelizzola, Benchmarking of computational methods for m^6^A profiling with Nanopore direct RNA sequencing. Brief. Bioinform. 25, bbae001 (2024). 38279646 10.1093/bib/bbae001 PMC10818168

[64] Z.-D. Zhong, Y.-Y. Xie, H.-X. Chen, Y.-L. Lan, X.-H. Liu, J.-Y. Ji, F. Wu, L. Jin, J. Chen, D. W. Mak, Z. Zhang, G.-Z. Luo, Systematic comparison of tools used for m6A mapping from nanopore direct RNA sequencing. Nat. Commun. 14, 1906 (2023). 37019930 10.1038/s41467-023-37596-5 PMC10076423

[65] S. Cruciani, A. D. Tejedor, L. Pryszcz, R. Medina, L. Llovera, E. M. Novoa, De novo basecalling of RNA modifications at single molecule and single nucleotide resolution. Genome Biol. 26, 38 (2025). 40001217 10.1186/s13059-025-03498-6 PMC11853310

[66] W.-S. Liau, Q. Zhao, A. Bademosi, R. S. Gormal, H. Gong, P. R. Marshall, A. Periyakaruppiah, S. U. Madugalle, E. L. Zajaczkowski, L. J. Leighton, H. Ren, M. Musgrove, J. Davies, S. Rauch, C. He, B. C. Dickinson, X. Li, W. Wei, F. A. Meunier, S. M. Fernández-Moya, M. A. Kiebler, B. Srinivasan, S. Banerjee, M. Clark, R. C. Spitale, T. W. Bredy, Fear extinction is regulated by the activity of long noncoding RNAs at the synapse. Nat. Commun. 14, 7616 (2023). 37993455 10.1038/s41467-023-43535-1 PMC10665438

[67] C.-W. Wei, T. Luo, S.-S. Zou, A.-S. Wu, The role of long noncoding RNAs in central nervous system and neurodegenerative diseases. Front. Behav. Neurosci. 12, 175 (2018). 30323747 10.3389/fnbeh.2018.00175 PMC6172704

[68] Y. D. J. Prawer, J. Gleeson, R. D. Paoli-Iseppi, M. B. Clark, Pervasive effects of RNA degradation on Nanopore direct RNA sequencing. NAR Genom. Bioinform. 5, lqad060 (2023). 37305170 10.1093/nargab/lqad060 PMC10251640

[69] Y. Liao, G. K. Smyth, W. Shi, featureCounts: An efficient general purpose program for assigning sequence reads to genomic features. Bioinformatics 30, 923–930 (2013). 24227677 10.1093/bioinformatics/btt656

[70] J. Gleeson, A. Leger, Y. D. J. Prawer, T. A. Lane, P. J. Harrison, W. Haerty, M. B. Clark, Accurate expression quantification from nanopore direct RNA sequencing with NanoCount. Nucleic Acids Res. 50, e19 (2022). 34850115 10.1093/nar/gkab1129 PMC8886870

[71] M. E. Ritchie, B. Phipson, D. Wu, Y. Hu, C. W. Law, W. Shi, G. K. Smyth, *limma* powers differential expression analyses for RNA-sequencing and microarray studies. Nucleic Acids Res. 43, e47 (2015). 25605792 10.1093/nar/gkv007 PMC4402510

[72] C. W. Law, Y. Chen, W. Shi, G. K. Smyth, voom: Precision weights unlock linear model analysis tools for RNA-seq read counts. Genome Biol. 15, R29 (2014). 24485249 10.1186/gb-2014-15-2-r29 PMC4053721

[73] K. Vitting-Seerup, A. Sandelin, IsoformSwitchAnalyzeR: Analysis of changes in genome-wide patterns of alternative splicing and its functional consequences. Bioinformatics 35, 4469–4471 (2019). 30989184 10.1093/bioinformatics/btz247

[74] S. Anders, A. Reyes, W. Huber, Detecting differential usage of exons from RNA-seq data. Genome Res. 22, 2008–2017 (2012). 22722343 10.1101/gr.133744.111 PMC3460195

[75] X. Dong, M. R. M. Du, Q. Gouil, L. Tian, J. S. Jabbari, R. Bowden, P. L. Baldoni, Y. Chen, G. K. Smyth, S. L. Amarasinghe, C. W. Law, M. E. Ritchie, Benchmarking long-read RNA-sequencing analysis tools using in silico mixtures. Nat. Methods 20, 1810–1821 (2023). 37783886 10.1038/s41592-023-02026-3

[76] A. O. Olarerin-George, S. R. Jaffrey, MetaPlotR: A Perl/R pipeline for plotting metagenes of nucleotide modifications and other transcriptomic sites. Bioinformatics 33, 1563–1564 (2017). 28158328 10.1093/bioinformatics/btx002 PMC5860047

[77] T. Wu, E. Hu, S. Xu, M. Chen, P. Guo, Z. Dai, T. Feng, L. Zhou, W. Tang, L. Zhan, X. Fu, S. Liu, X. Bo, G. Yu, clusterProfiler 4.0: A universal enrichment tool for interpreting omics data. Innovation 2, 100141 (2021). 34557778 10.1016/j.xinn.2021.100141 PMC8454663

[78] S. X. Ge, D. Jung, R. Yao, ShinyGO: A graphical enrichment tool for animals and plants. Bioinformatics 36, 2628–2629 (2019). 10.1093/bioinformatics/btz931 PMC7178415 31882993

