# Supplementary material for: Isoform-level profiling of m6A epitranscriptomic signatures in human brain
Source: Sci Adv. 2025 Aug 8;11(32):eadp0783. doi: 10.1126/sciadv.adp0783 (PMC12333690; doi:10.1126/sciadv.adp0783)
Supplement: Supplementary file 1 — Figs. S1 to S8 Tables S1 and S2 Legends for tables S3 to S15 [file sciadv.adp0783_sm.pdf]

Science Advances
AAAS

Supplementary Materials for

Isoform-level profiling of m^{6}A epitranscriptomic signatures in human brain

Josie Gleeson *et al.*

Corresponding author: Michael B. Clark, michael.clark@unimelb.edu.au;
Ricardo De Paoli-Iseppi, ric.depaoliiseppi@unimelb.edu.au

Sci. Adv. **11**, eadp0783 (2025)
DOI: 10.1126/sciadv.adp0783

The PDF file includes:

Figs. S1 to S8
Tables S1 and S2
Legends for tables S3 to S15

Other Supplementary Material for this manuscript includes the following:

Tables S3 to S15

Supplementary Materials

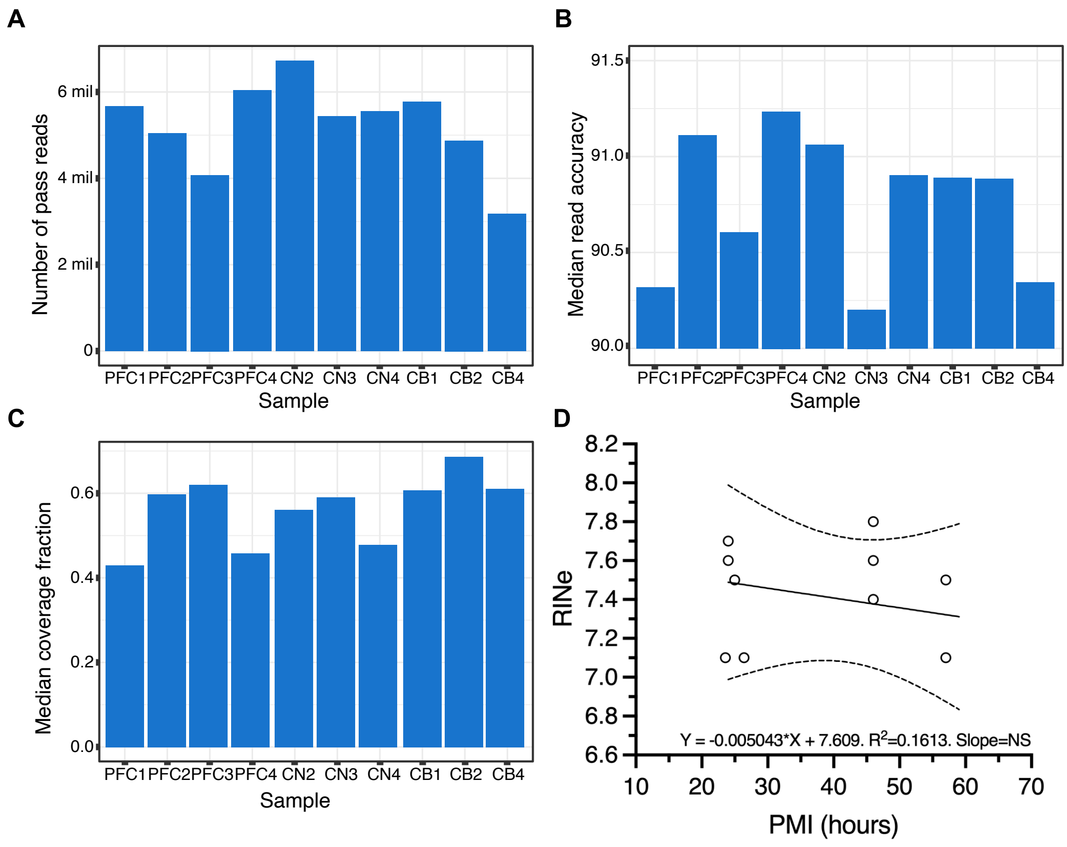

Supplementary Figure 1. Metrics for direct RNA sequencing of 10 samples.

(**A**) Total number of pass (qscore>7) reads per sample. (**B**) Median read accuracy per sample calculated from CIGAR strings in BAM files as: (M+I+D-NM)/(M+I+D). (**C**) Median coverage fraction of reads per sample calculated as: alignment length / mapped isoform length. (**D**) Post-mortem interval (hours) per sample compared with sample RNA integrity number (RIN).

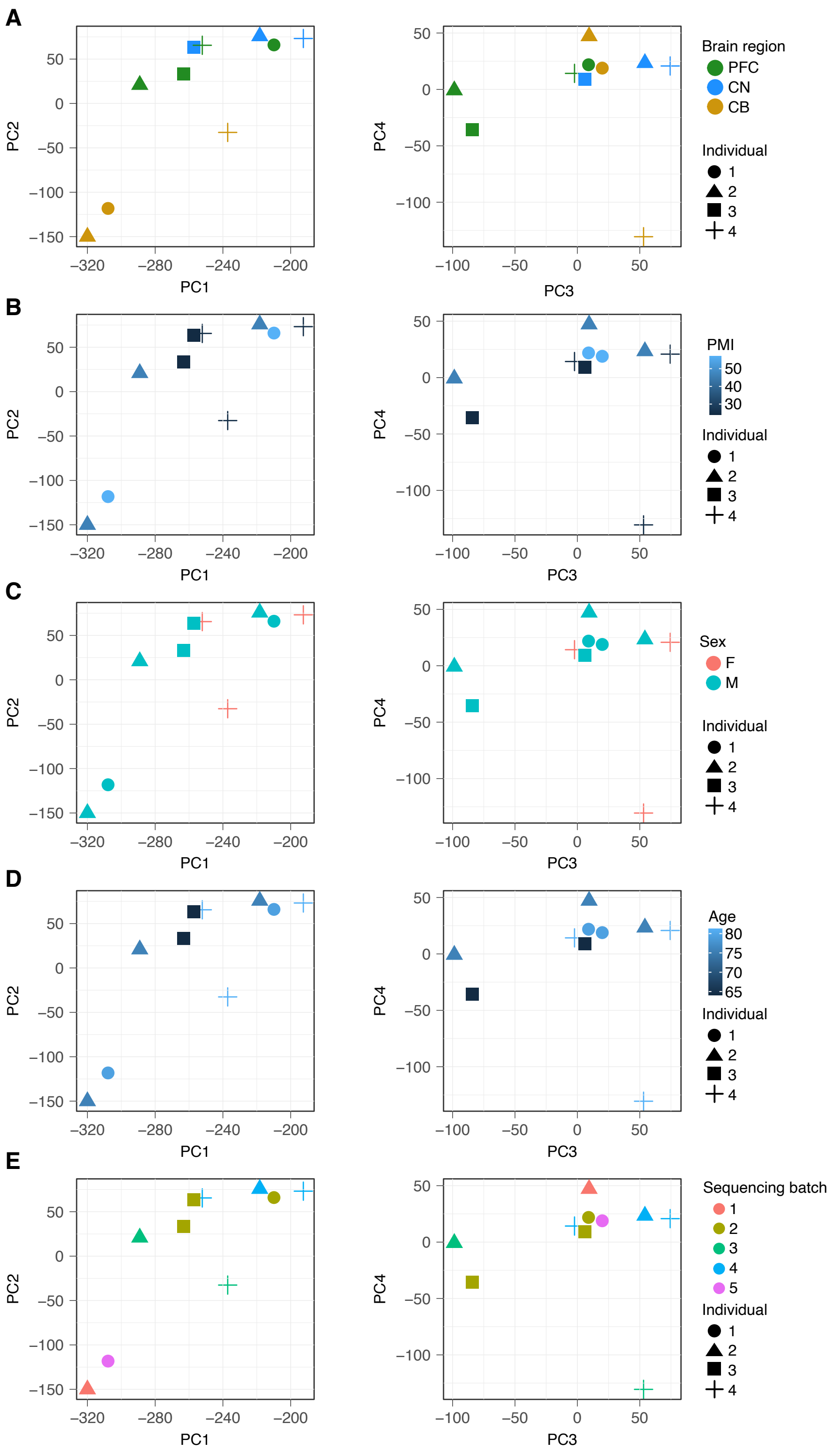

Supplementary Figure 2. PCAs of m6A modification rates per sample.

Plots show PC1 vs PC2 (left) and PC3 vs PC4 (right) with shapes representing the individual donor ID. Plots are coloured by (**A**) sample brain region, (**B**) donor post-mortem interval (PMI) hours, (**C**) donor sex, (**D**) donor age, (**E**) RNA sequencing batch.

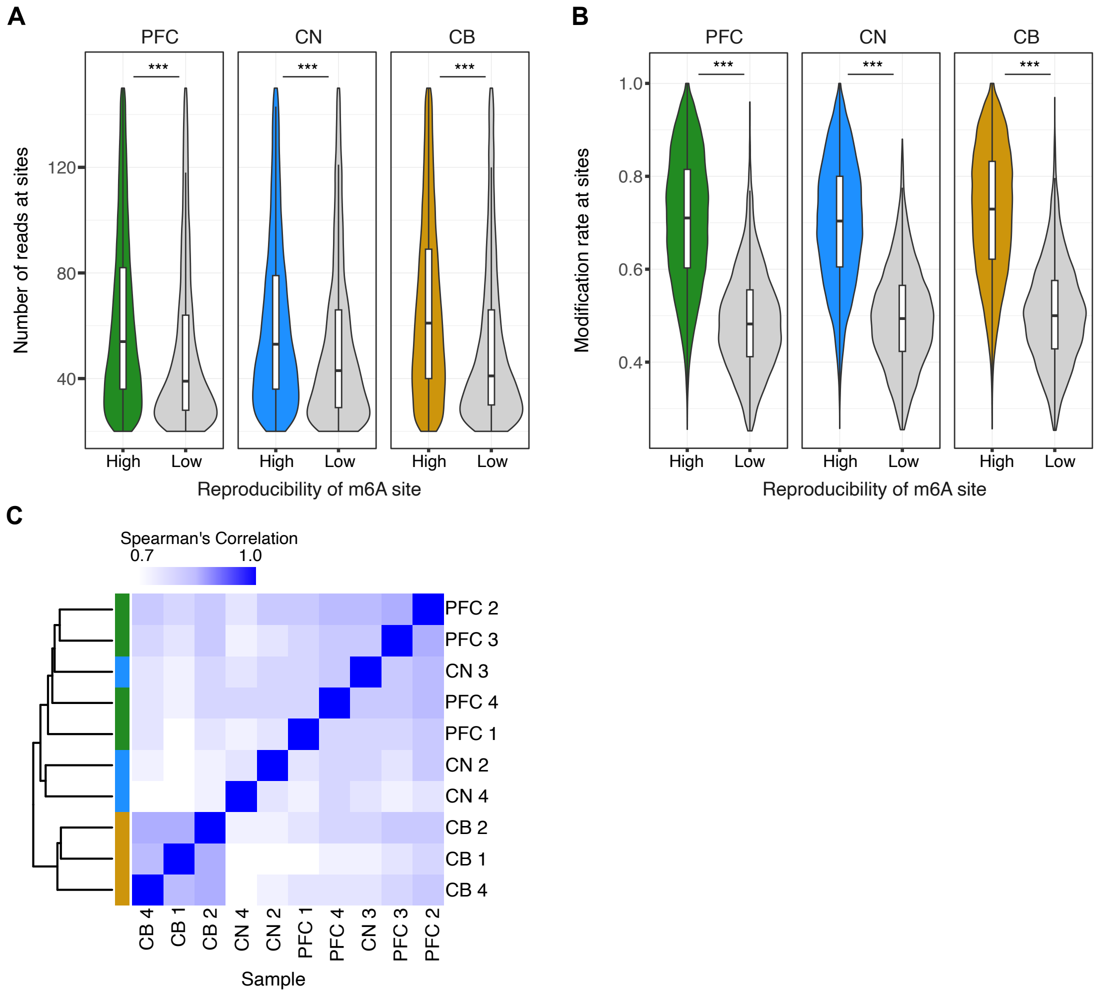

Supplementary Figure 3. Read coverages and modification rates at m6A sites.

(**A**) The number of reads at m6A sites and, (**B**) modification rates at m6A sites that were classified as either high or low reproducibility. High: detected in at least 3 samples per brain region. Low: detected in two or less samples per brain region. (**C**) Heatmap of correlations between modification rates of m6A sites per sample.

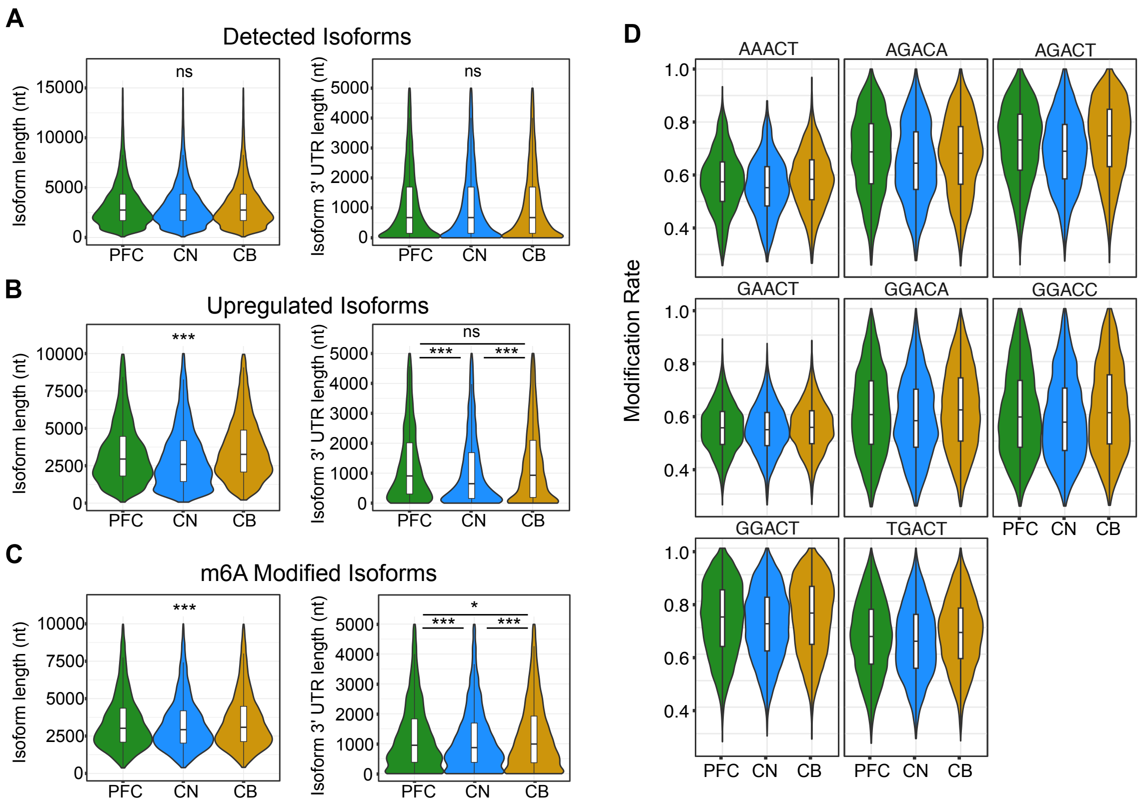

Supplementary Figure 4. Isoform lengths, 3'UTR lengths and modification rates in each brain region.

The isoform lengths (left plots in panels **A-C**) and 3' UTR lengths (right plots in panels **A-C**) were compared between brain regions for different isoform sets. (**A**) Detected isoforms (counts >5) showed no significant differences between brain regions. (**B**) Isoforms that were upregulated (significantly differentially expressed) in their respective brain regions showed significant differences in length between all brain regions, and significantly longer 3'UTRs in PFC and CB compared to CN. (**C**) Isoforms that were m6A modified in each of the respective brain regions showed significant differences in both isoform length and 3'UTR length between all brain regions. P-values from Mann-Whitney-Wilcoxon tests are indicated by 'ns' for >0.05, '*' for <0.05 and '***' for <0.001, where only one value is shown, all comparisons had this consistent value. (**D**) Modification rates at m6A sites plotted for each DRACH motif per brain region.

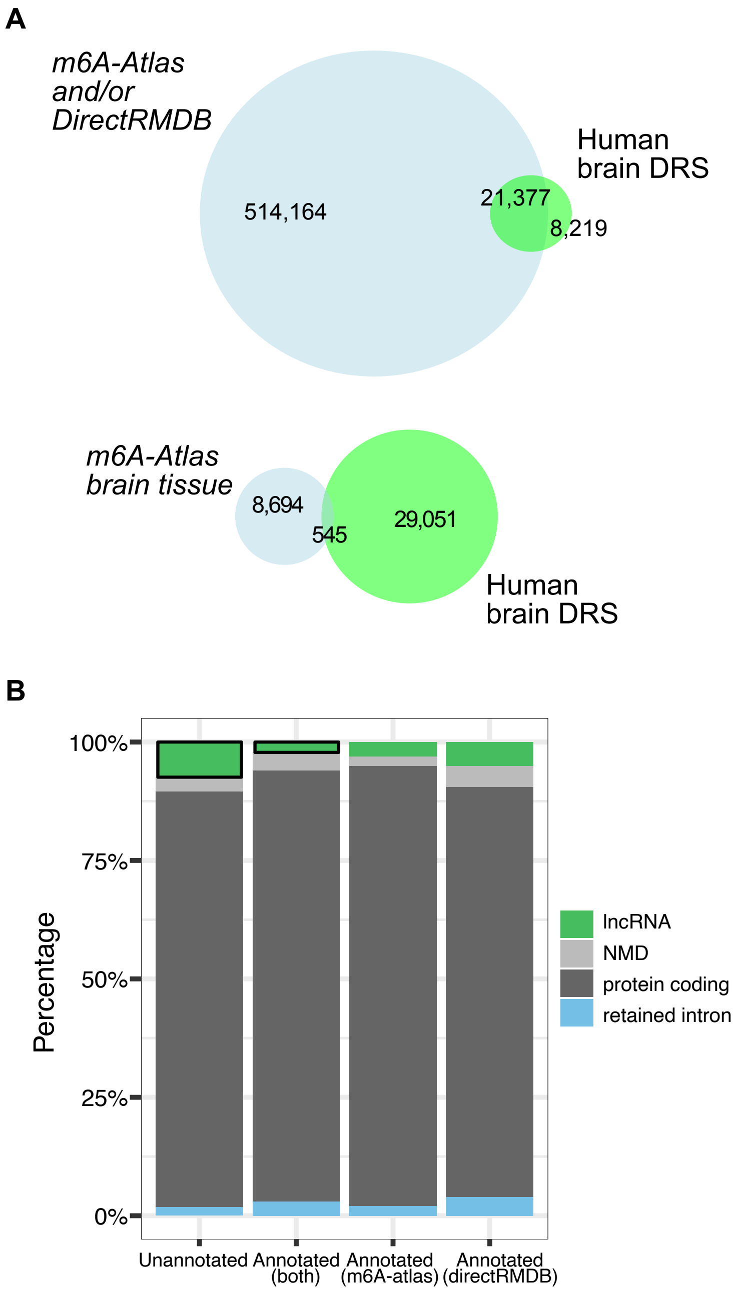

Supplementary Figure 5. Unannotated vs annotated m6A sites.

(**A**) Venn diagrams showing intersections of m6A sites identified in our human brain DRS data compared with (top) those identified in either m6A-Atlas or DirectRMDB and (bottom) m6A-Atlas sites from human brain tissue (27,28). (**B**) A higher percentage of previously unannotated m6A sites are within lncRNAs compared with those annotated in both *DirectRMDB* and/or *m6A-Atlas* (boxes with black outlines indicate lncRNA categories in each group). Unannotated lncRNA = 7.4%, annotated in both databases lncRNA = 2.2%, p-value<0.0001 (two-proportion z-test).

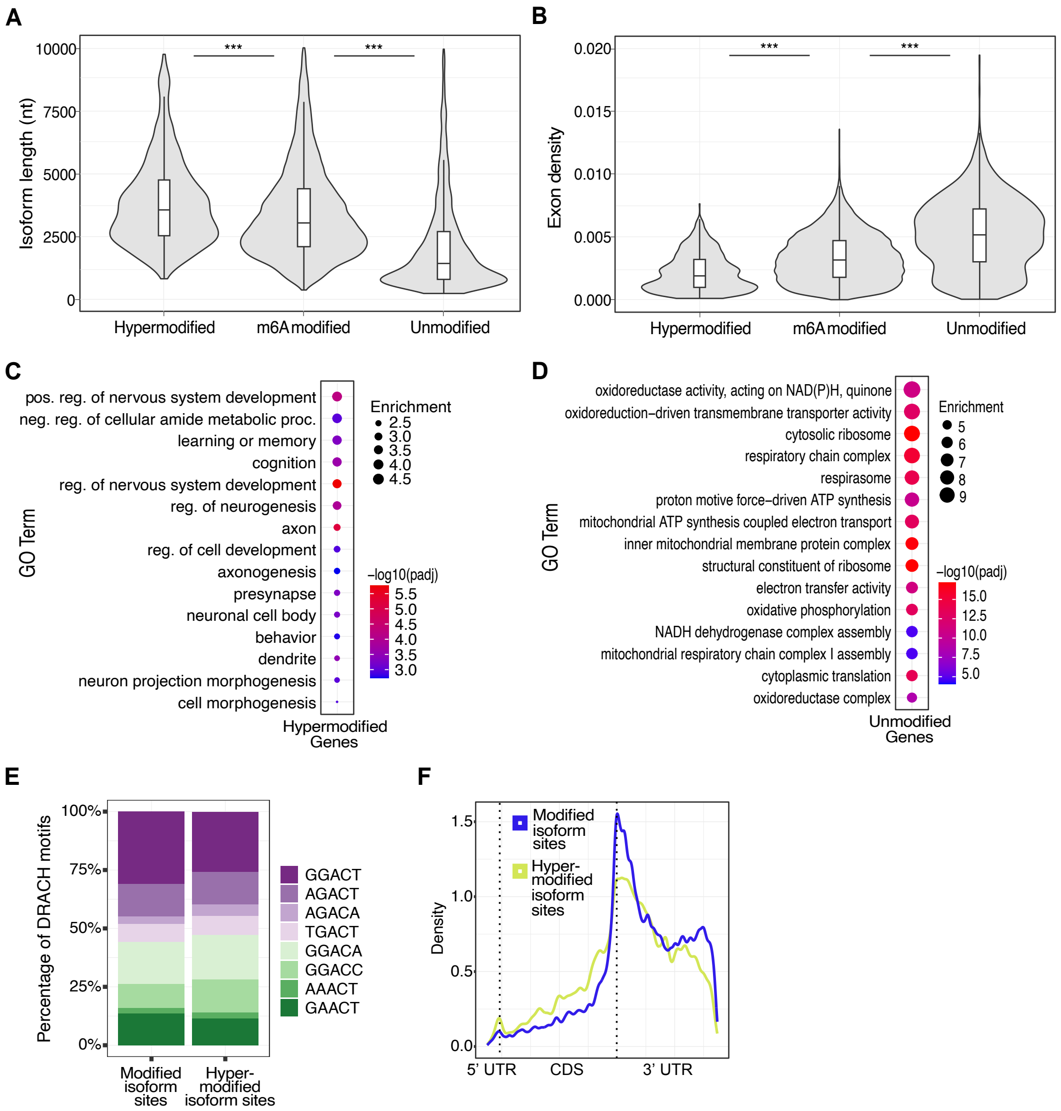

Supplementary Figure 6. Comparison of hypermodified, modified and unmodified isoforms.

(**A,B**) Hypermodified, modified and unmodified isoforms were compared for (**A**) isoform length (nt) and (**B**) exon density per isoform (exon density = number of exons / isoform length). Significance of Mann-Whitney U p-values is indicated by '***' for <0.001. (**C**) Gene ontology (GO) analysis of genes from 911 hypermodified isoforms. (**D**) GO analysis of genes from 3,907 unmodified isoforms. (**E**) Percentages of each DRACH motif in all m6A sites and m6A sites within hypermodified isoforms. (**F**) Metagene plot showing the distribution of all m6A sites and m6A sites in hypermodified isoforms (Kolmogorov–Smirnov p<0.0001).

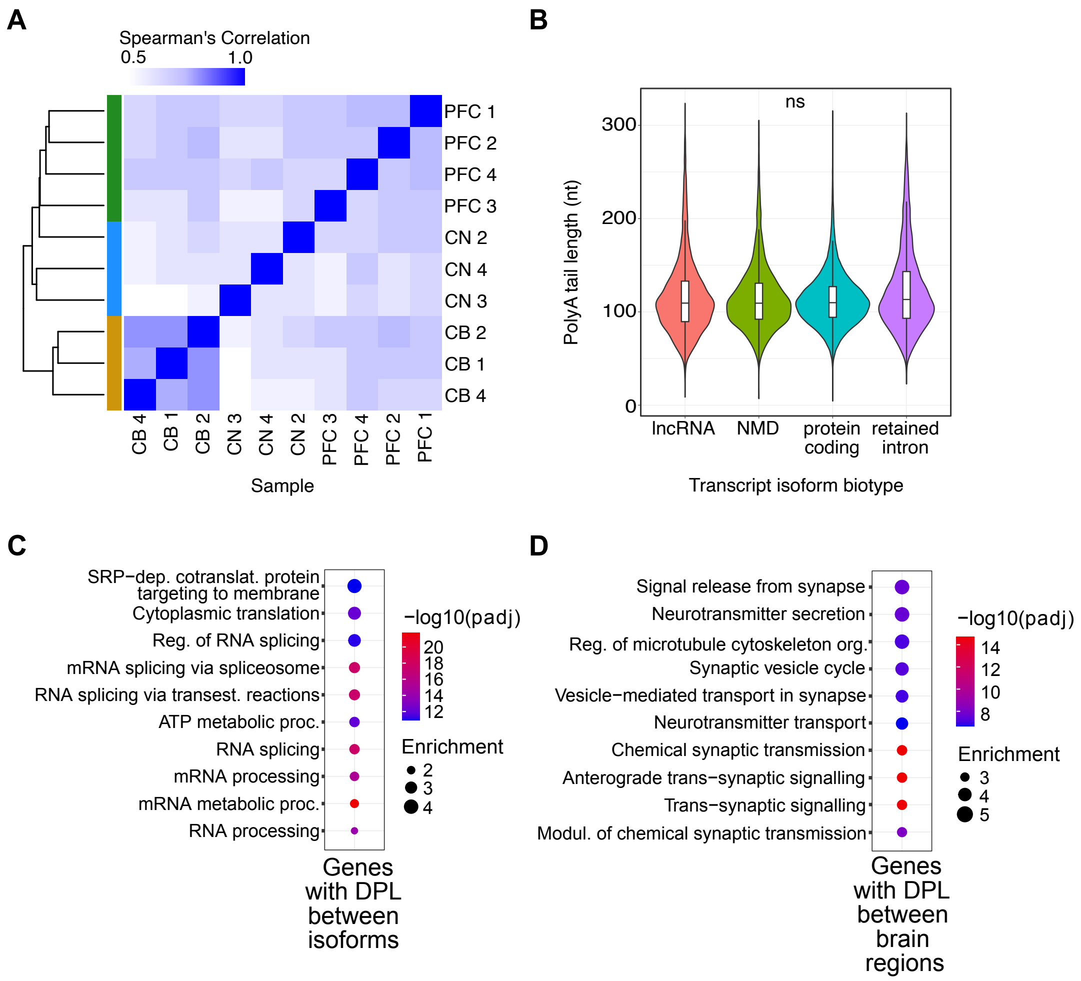

Supplementary Figure 7. PolyA length comparisons and GO analysis.

(**A**) Heatmap showing that samples from the same brain region largely clustered together based on the median polyA length per isoform. (**B**) Violin plots of polyA lengths between different transcript isoform biotypes. No significant differences in polyA lengths were observed. (**C,D**) GO analysis of genes with (**C**) differential polyA lengths (DPL) between isoforms in a single brain region and (**D**) DPL between the same isoform in different brain regions.

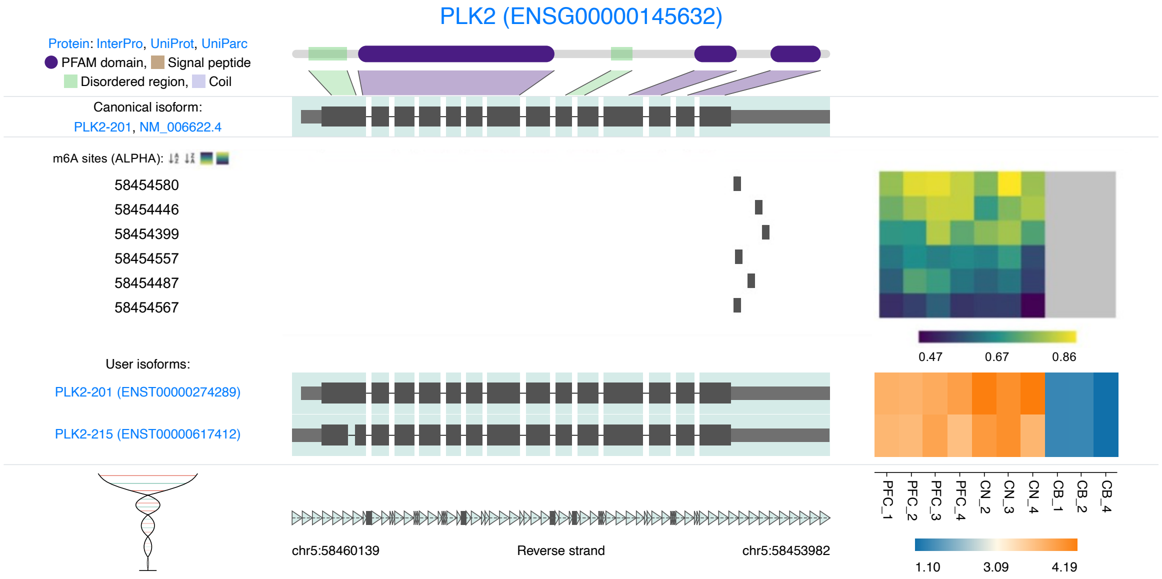

Supplementary Figure 8. Visualisation of m6A modification sites with IsoVis.

Example using the *PLK2* gene of the data exploration and visualisation of m6A modification sites and rates with the IsoVis webserver. Users can upload isoform coordinates (BED or GTF file), isoform expression data (CSV file), m6A modification positions (BED file) and m6A modification rates (CSV file). The ENSEMBL canonical isoform is shown at the top with protein domains annotated as coloured boxes. Genomic locations of user uploaded m6A sites are displayed below and labelled by their genomic coordinates (left hand side), with m6A modification rates shown in a heatmap (right hand side). The mean modification rate was calculated per genomic position per sample. Samples with no evidence of an m6A modification or no data are shown as grey in the heatmap. The bottom track shows expressed isoforms present in user data, with their expression values displayed in a heatmap.

Supplementary Table 1. Direct RNA sequencing metrics of 10 post-mortem human brain samples from 4 individual donors.

	PFC 1	PFC 2	PFC 3	PFC 4	CN 2	CN 3	CN 4	CB 1	CB 2	CB 4	Total
Total pass reads (millions)	5.67	5.05	4.08	6.04	6.73	5.44	5.56	5.78	4.88	3.18	52.4
Median read alignment length (nt)	656	792	803	656	643	687	580	753	919	756	720
Median coverage fraction of reads	0.43	0.60	0.62	0.46	0.56	0.59	0.48	0.61	0.69	0.61	0.59
Median accuracy of reads (%)	90.30	91.10	90.59	91.23	91.05	90.20	90.89	90.83	90.81	90.31	90.82
Number of genes identified (count>5)	15,311	14,815	14,052	15,051	14,989	14,921	15,025	15,077	14,786	13,389	22,608
Number of isoforms identified (count>5)	33,501	31,420	28,534	32,890	32,055	31,271	32,032	33,153	32,078	27,104	62,577

Supplementary Table 2. Sample metadata.

	PFC 1	PFC 2	PFC 3	PFC 4	CN 2	CN 3	CN 4	CB 1	CB 2	CB 4
Individual	1	2	3	4	2	3	4	1	2	4
RNA integrity number	7.5	7.6	7.6	7.5	7.4	7.7	7.1	7.1	7.8	7.1
Age	79.3	75.6	64.1	81.2	75.6	64.1	81.2	79.3	75.6	81.2
Sex	M	M	M	F	M	M	F	M	M	F
Post-mortem interval (hours)	57	46	24	25	46	24	25	57	46	25
pH	6.63	6.57	6.56	6.4	6.57	6.56	6.4	6.63	6.57	6.4
Weight (mg)	257	230	243	224	203	254	216	261	250	248
RNA extraction batch	1	4	2	3	2	3	4	3	1	4
Sequencing batch	2	3	2	4	4	2	4	5	1	3

Supplementary Table 3. Differentially expressed genes (DEGs), isoforms (DEIs) and differential isoform usage (DIU) between brain regions.

Supplementary Table 4. GO analysis of genes with DIU, DEI or identified as DEGs.

Supplementary Table 5. All and filtered high-confidence m6A sites identified by m6Anet.

Supplementary Table 6. Differential gene expression results for m6A machinery-related genes.

Supplementary Table 7. GO enrichments for annotated and unannotated m6A sites.

Supplementary Table 8. Isoforms specifically modified in a brain region without evidence of expression upregulation in that brain region.

Supplementary Table 9. GO and cell-type enrichment of isoforms with region-specific m6A modification.

Supplementary Table 10. Hypermodified and unmodified isoforms.

Supplementary Table 11. GO and cell-type enrichment of hypermodified and unmodified isoforms.

Supplementary Table 12. Genomic sites with differential m6A modification rates between different gene isoforms.

Supplementary Table 13. Identity and GO enrichments of isoforms with differentially modified m6A sites between brain regions.

Supplementary Table 14. Isoforms with dynamic polyA tail lengths. Differential polyA lengths (DPL) between isoforms and brain regions.

Supplementary Table 15. GO enrichments for isoforms with dynamic polyA tail lengths and differential polyA lengths.
